# A highly potent lymphatic system–targeting nanoparticle cyclosporine prevents glomerulonephritis in mouse model of lupus

**DOI:** 10.1126/sciadv.abb3900

**Published:** 2020-06-12

**Authors:** Raghu Ganugula, Meenakshi Arora, Dianxiong Zou, Sandeep K. Agarwal, Chandra Mohan, M. N. V. Ravi Kumar

**Affiliations:** 1 Department of Pharmaceutical Sciences, Irma Lerma Rangel College of Pharmacy, Texas A&M University, College Station, TX, USA.; 2 Section of Immunology, Allergy and Rheumatology, Department of Medicine, Biology of Inflammation Baylor College of Medicine, One Baylor Plaza, Houston, TX, USA.; 3 Department of Biomedical Engineering, University of Houston, Houston, TX, USA.

## Abstract

Cyclosporine A (CsA) is a powerful immunosuppressant, but it is an ineffective stand-alone treatment for systemic lupus erythematosus (SLE) due to poor target tissue distribution and renal toxicity. We hypothesized that CD71 (transferrin receptor 1)–directed delivery of CsA to the lymphatic system would improve SLE outcomes in a murine model. We synthesized biodegradable, ligand-conjugated nanoparticles [P2Ns–gambogic acid (GA)] targeting CD71. GA conjugation substantially increased nanoparticle association with CD3^+^ or CD20^+^ lymphocytes and with intestinal lymphoid tissues. In orally dosed MRL-*lpr* mice, P2Ns-GA–encapsulated CsA increased lymphatic drug delivery 4- to 18-fold over the ligand-free formulation and a commercial CsA capsule, respectively. Improved lymphatic bioavailability of CsA was paralleled by normalization of anti–double-stranded DNA immunoglobulin G titer, plasma cytokines, and glomerulonephritis. Thus, this study demonstrates the translational potential of nanoparticles that enhance the targeting of lymphatic tissues, transforming CsA into a potent single therapeutic for SLE.

## Teaser

Orally delivered lymphatic system–specific nanoparticle cyclosporine is an effective way to treat lupus.

## INTRODUCTION

Systemic lupus erythematosus (SLE) is a chronic inflammatory disease characterized by the loss of central and peripheral tolerance, emergence of autoantibodies, accumulation of immune complexes, and extensive damage to vital tissues from infiltrating leukocytes (*1*–*3*). SLE exhibits a broad range of manifestations in virtually every organ, from cutaneous flares to life-threatening glomerulonephritis (*3*). Because the disease cannot be cured, standard SLE treatments have relied on long-term management with a combination of NSAIDs (nonsteroidal anti-inflammatory drugs), corticosteroid, antimalarial, and cytostatic drugs (*4*). However, the existence of refractory SLE and numerous side effects of continuous immunosuppressant use (*5*, *6*) necessitate the research of alternative therapies. On the basis of the known etiology of SLE (*1*, *2*), an effective drug should simultaneously suppress the proliferation or activity of autoreactive lymphocytes while sparing the homeostatic functions of the immune system. Some of the antibody-based therapies, such as belimumab (anti–B cell–activating factor), that target the maladaptive signaling components of B cells are promising (*7*, *8*), but the development of many biologics targeting SLE T cells (e.g., abatacept, ruplizumab, and rontalizumab) has been challenged by poor clinical trial outcomes (*7*).

Cyclosporine A (CsA) is a fungal-derived cyclic peptide that has been routinely prescribed to suppress organ rejection in allograft recipients (*9*). The core mechanism underlying CsA pharmacology involves the cyclophilin-dependent inhibition of calcineurin. The formation of a high-affinity calcineurin/drug complex results in approximately 1000-fold lower dephosphorylation and nuclear translocation of nuclear factor of activated T cells (NFAT) (*9*), thereby interfering with the transcription of genes necessary for T cell activation and survival [e.g., interleukin-2 (*IL-2*), *IL-4*, *IL-6*, and interferon-γ (*IFN*-γ)], as well as for T cell–dependent B cell maturation (e.g., *CD40L*) (*10*). In vitro, CsA has also been shown to negatively regulate the functions of other leukocytes, such as mast cells, neutrophils, natural killer cells, and mononuclear phagocytes, primarily by attenuating their Ca^++^-dependent response to pro-inflammatory stimuli (*11*–*13*). These broad immunosuppressive capabilities make CsA an appealing candidate to treat SLE. Off-label use of CsA in conjunction with corticosteroids was met with moderate success in treating lupus nephritis while allowing the added benefit of steroid sparing (*14*). Moreover, other calcineurin inhibitors, such as tacrolimus and voclosporin, have been tested in patients with lupus with positive outcomes (*15*). No study to date, however, has demonstrated the efficacy of calcineurin inhibitor as a stand-alone treatment for lupus. The narrow therapeutic index of CsA, caused by its low and variable target tissue bioavailability, coupled with high nephrotoxicity (*9*), poses a major, unresolved challenge that limits the translational potential of this potent drug.

Our laboratory has found that nanoparticle encapsulation of CsA can significantly enhance its oral delivery and tissue disposition in the healthy rat model (*16*, *17*). The nanoparticle embodiment, called P2Ns–gambogic acid (GA), consists of a biodegradable, spherical capsule of polylactide (PLA)/polyethylene glycol (PEG) polymers that, in turn, are periodically functionalized with carboxyl groups, each of which conjugated to GA via an ethylenediamine linker (*17*). This configuration allows the flexible, outward orientation of GA, a ligand (*16*, *17*) targeting CD71 (transferrin receptor 1), which is expressed by the intestinal epithelia, intervillous crypts, and Peyer’s patches (*18*) as well as by mature and precursor lymphocytes (*19*). The GA-CD71 binding likely occurs at the extracellular protease-like domain of CD71 (*20*) and facilitates the clathrin-dependent, receptor-mediated endocytosis of nanoparticles (*16*, *17*). Compared to unconjugated nanoparticles (P2Ns), P2Ns-GA exhibits better trafficking across the gastrointestinal barrier (*17*) and potentially improves transcytosis into the intestinal capillary network or gut-associated lymphatic tissue (GALT). Because SLE is an immunologically driven disease (*1*, *2*), increased delivery of a potent immunosuppressant such as CsA to the systemic lymphatic circulation through the GALT should provide therapeutic benefit. Here, we used mouse peripheral blood mononuclear cells (PBMCs) and a murine model (MRL-*lpr*) with aggressive lupus-like disease (*21*) to address the questions of lymphatic targeting and the efficacy of various CsA formulations.

## RESULTS

### FACS analysis of nanoparticle binding to PBMCs

**Fig. 1:**
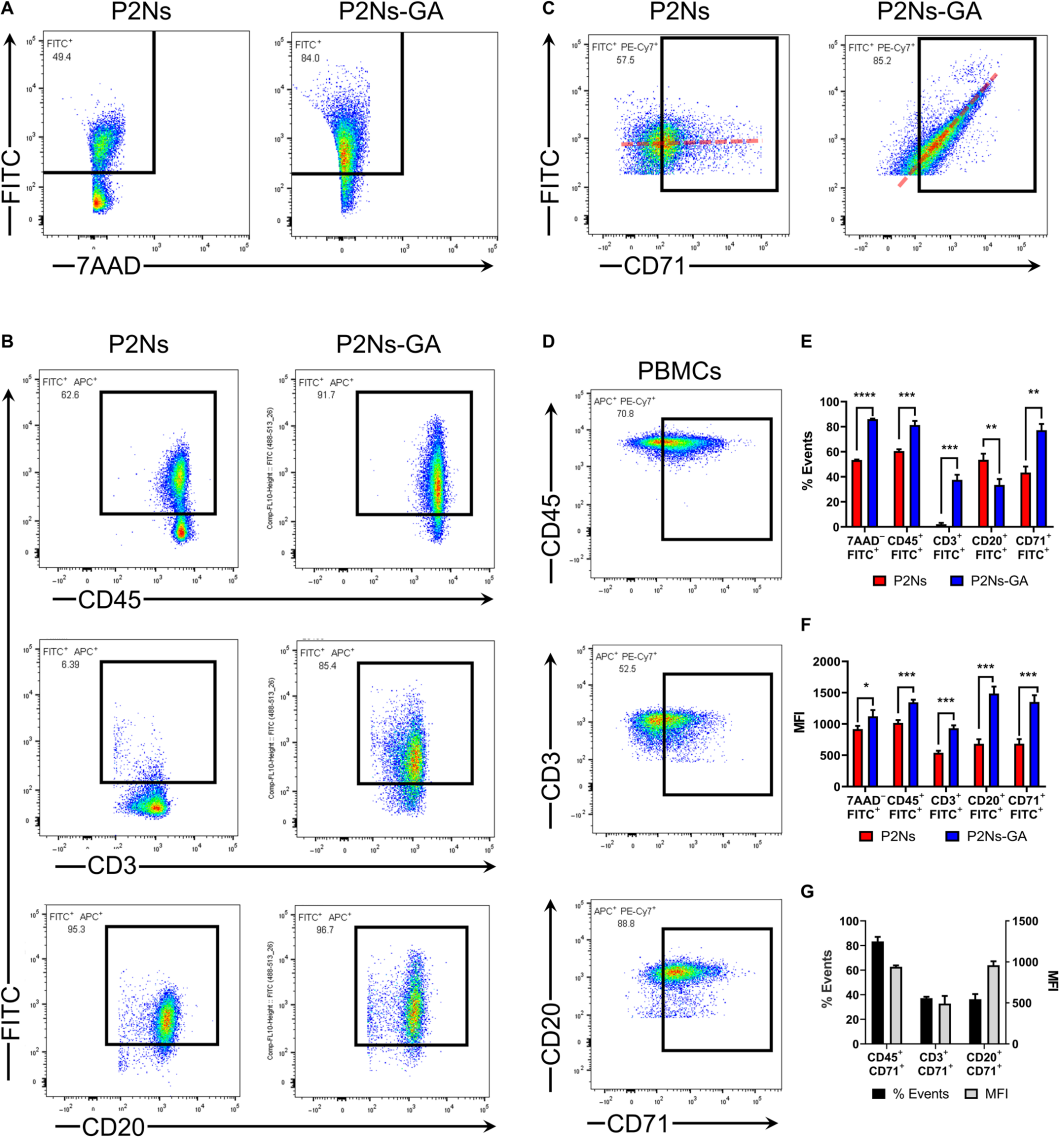
FACS analysis of P2Ns/P2Ns-GA binding in PBMCs. (**A**) Population of murine PBMCs displaying the fluorescence intensities of P2Ns/P2Ns-GA binding (FITC) and 7-amino-actinomycin D (7AAD) staining. (**B**) P2Ns/P2Ns-GA binding (FITC) among mononuclear leukocyte subsets: CD45 (common leukocyte antigen), CD3 (T cell marker), and CD20 (B cell marker). (**C**) P2Ns/P2Ns-GA binding (FITC) as a function of CD71 surface expression showed that the association of P2Ns with PBMCs was not correlated with CD71 expression, whereas the association of P2Ns-GA was linearly correlated (i.e., dotted red lines overlaying both plots). (**D**) CD71 surface expression among various mononuclear leukocyte subsets. (**E**) Graphical representations of % events (FITC^+^ signals) for (A) to (C). (**F**) Graphical representations of MFI (FITC^+^ signals) for (A) to (C). (**G**) Graphical representations of % events and MFI (CD71^+^ signals) for (D). *n* = 3 for all data, from individual experiments performed on different days. Data are represented as mean ± SEM. *P < 0.05, **P < 0.01, ***P < 0.001, and ****P < 0.0001; ns, not significant; comparisons were made with two-tailed Student’s *t* test.

We performed in vitro binding assays (*17*) with PBMCs isolated from healthy Institute of Cancer Research (ICR) mice to determine whether the association of nanoparticles with lymphocytes was influenced by GA conjugation. Incubation of fluorescent [fluorescein isothiocyanate (FITC)] P2Ns-GA or P2Ns (ligand-free nanoparticle) with PBMCs in serum-free media resulted in robust binding for both groups. However, the fraction of cells with FITC signal (% events) and the median FITC intensity (MFI) per cell were significantly higher for P2Ns-GA (Fig. 1, A, E, and F). We further studied the lymphocyte subpopulations that underwent differential nanoparticle binding by labeling the PBMCs with anti-CD3 (T cell lineage marker), anti-CD20 (B cell lineage marker), or anti-CD45 (common leukocyte marker) fluorescent APC (allophycocyanin) antibodies. Compared P2Ns, P2Ns-GA showed higher FITC % events and MFI for both CD45^+^ and CD3^+^ subsets, whereas higher MFI, but similar % events were observed for the CD20^+^ subset (Fig. 1, B, E, and F). The changes in binding between the two nanoparticles were most notable in the CD3^+^ subset, which exhibited a sixfold increase in % events with P2Ns-GA.

To determine the relevance of CD71 in lymphatic transport, we labeled PBMCs with anti-CD71 fluorescent PE (phycoerythrin)–Cy7 antibody and found that compared to P2Ns, P2Ns-GA was more abundantly associated with CD71^+^ cells (Fig. 1, C, E, and F). Moreover, the magnitude of P2Ns-GA binding (FITC intensity) in any particular cell appeared to be directly correlated with the degree of CD71 protein expression (PE-Cy7 intensity), whereas no such correlation existed for P2Ns-treated PBMCs (Fig. 1C). Co-labeling PBMCs with the anti-CD71 antibody and anti-CD3, CD20, or CD45 antibodies revealed that CD71 was expressed in a significant proportion of mononuclear leukocytes, including B and T cells, with CD20^+^ B cells expressing more CD71 (MFI and PE-Cy7 fluorescence) than CD3^+^ T cells (Fig. 1, D and G). The higher CD71 expression in B cells compared to T cells had been indirectly confirmed by transcriptome-wide datasets (GSE10325 and GSE4588) (*22*), and such difference may account for greater P2Ns-GA binding (MFI) among B cells.

### Confocal microscopy of PBMCs

**Fig. 2:**
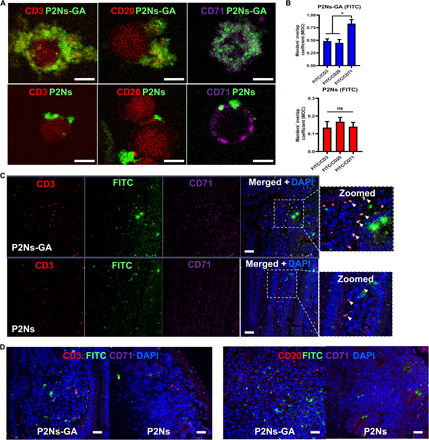
In vitro and ex vivo association of P2Ns/P2Ns-GA with PBMCs and MRL-*lpr* intestinal tissue sections. (**A**) Confocal imaging of mouse PBMCs in vitro association with fluorescent (green) P2Ns-GA or P2Ns. GA conjugation, which targets CD71 (purple), resulted in more robust nanoparticle association with CD3^+^ T cells (red) and CD20^+^ B cells (red) (scale bars, 5 μm, for all images). (**B**) Manders’ overlap coefficient (MOC; a measurement of fluorescent signal colocalization) showed greater signal overlap between P2Ns-GA and CD71, compared to either CD3 or CD20. In contrast, P2Ns showed no preferential overlap with CD71, CD3, or CD20 (*n* = 4 images). (**C**) Ex vivo, deparaffinized tissue sections from MRL-*lpr* mouse small intestine appeared to show more P2Ns-GA infiltration of lacteals (top) and (**D**) Peyer’s patches compared to P2Ns. Furthermore, triple colocalization of nanoparticles, CD3, and CD71 was qualitatively observed for P2Ns-GA [(C), top, zoomed image with arrowheads], whereas no such triple colocalization was apparent in the P2Ns ex vivo assay [(C), bottom, zoomed image with arrowheads] (scale bars, 50 μm, for all images). Representative images are shown. Data are represented as mean ± SEM. *P < 0.05; comparisons were made with one-way analysis of variance (ANOVA), followed by Tukey’s multiple comparisons test.

After quantifying the effects of P2Ns-GA on bulk PBMC populations, we proceeded to examine nanoparticle/lymphocyte interactions at the cellular level. Multiplex confocal laser scanning microscopy of formalin-fixed PBMCs was then performed to capture the localization of P2Ns and P2Ns-GA, alongside a host of immunological markers. We found that qualitatively, FITC-labeled P2Ns-GA was distributed throughout the cell, whereas P2Ns were mainly restricted to the cell surface (Fig. 2A). Co-staining PBMCs with anti-CD3 or CD20 antibodies, together with anti-CD71 antibody, revealed a generally weak pattern of colocalization between FITC and CD3 or CD20, respectively (Fig. 2B). Relative to CD3 and CD20, CD71 exhibited better colocalization with FITC-labeled P2Ns-GA than with P2Ns, based on Manders’ overlap coefficient (Fig. 2B). Along with fluorescence-activated cell sorting (FACS) analysis, the similar spatial distribution of CD71 and P2Ns-GA implies that CD71 likely plays a role in nanoparticle binding in a GA-dependent manner.

### Ex vivo nanoparticle binding to intestinal tissue

We have previously reported that GA conjugation enables greater absorption of nanoparticles by the epithelial mucosa and facilitated transport across the intestinal barrier (*16*, *17*). However, it was important to determine whether gut absorption could also involve the lacteals and Peyer’s patches underlying the epithelial mucosa, as they were constituents of the GALT, and provided a direct route for nanoparticles to enter lymphatic circulation (*23*). Using ex vivo jejunum sections from MRL-*lpr* mice, we incubated each section with P2Ns or P2Ns-GA, followed by antibodies against CD3 or CD20, and CD71. When focused on the intestinal villi, we observed that the underlying lamina propria was replete with CD3^+^ signals, which was consistent with the presence of lacteals and lymphatic circulation (Fig. 2C). In addition, these regions stained positive for both P2Ns and P2Ns-GA, but the FITC signal appeared stronger from P2Ns-GA (Fig. 2C). We also observed colocalization of P2Ns-GA (but not P2Ns) with CD71 at many of the CD3^+^ loci, implying that CD71/GA interaction could augment nanoparticle association with T lymphocyte–enriched regions (Fig. 2C). Peyer’s patches, which were small lymphoid nodules decorating the intestinal mucosa and whose endogenous function was to mediate immunosurveillance of pathogenic stimuli (*24*), also associated more with P2Ns-GA than P2Ns (Fig. 2D). However, perhaps owing to the high density of antigen-phagocytosing M cells present (*24*), we observed much higher nanoparticle association with Peyer’s patch regions that appeared to be independent of CD3/CD20 and CD71.

### Cyclosporine formulations used in the murine lupus model

In humans, the expansion of apoptosis-deficient, autoreactive T and B cells constitutes a major etiological driver of SLE (*1*, *2*), and a close analog of this disease is found in the lymphoproliferative MRL-*lpr* mice (*21*). CsA, the immunosuppressant we chose to modulate autoimmunity, was formulated in two different nanoparticles: P2Ns and P2Ns-GA, to obtain P2Ns-CsA and P2Ns-GA-CsA, respectively (fig. S1). These, along with a commercially available capsule form of generic Neoral (henceforth referred to as “CsA”), compose of the oral treatments used in this study. We selected an oral dose (5 mg/kg; CsA equivalent) for both nanoparticle and generic CsA treatments to minimize the likelihood of drug-related nephrotoxicity, which had been reported at dosages exceeding 20 mg/kg per day (*25*). To assess possible treatment or side effects without the active pharmaceutical ingredient, CsA-void P2Ns-GA was administered to naïve, MRL-*lpr* mice for the same study duration, but no major pathological changes were observed (fig. S2). Both drug-treated and untreated MRL-*lpr* mice were evaluated against the nonautoimmune MRL wild type on a 10-week dosing protocol.

### Hypertrophy of lymphoid organs

Organ-level lymphoproliferation is one of the most prominent hallmarks of SLE-like MRL-*lpr* mice (*21*). Those receiving no drug (MRL-*lpr* group) exhibited peripheral lymphadenopathy in the course of the study and upon sacrifice (fig. S3, A and C). The CsA treatment was unable to achieve a statistically significant reduction in lymph node size compared to MRL-*lpr*, whereas both nanoparticle CsA treatments were. The same efficacy pattern was observed for the mitigation of splenomegaly, and thymic hyperplasia was significantly attenuated by only CsA and P2Ns-GA-CsA (fig. S3, B, D, and E). Therefore, of the three lymphoproliferation pathologies examined at the organ level, only P2Ns-GA-CsA treatment was effective at addressing all three.

### Drug distribution and diagnostic panels

**Fig. 3:**
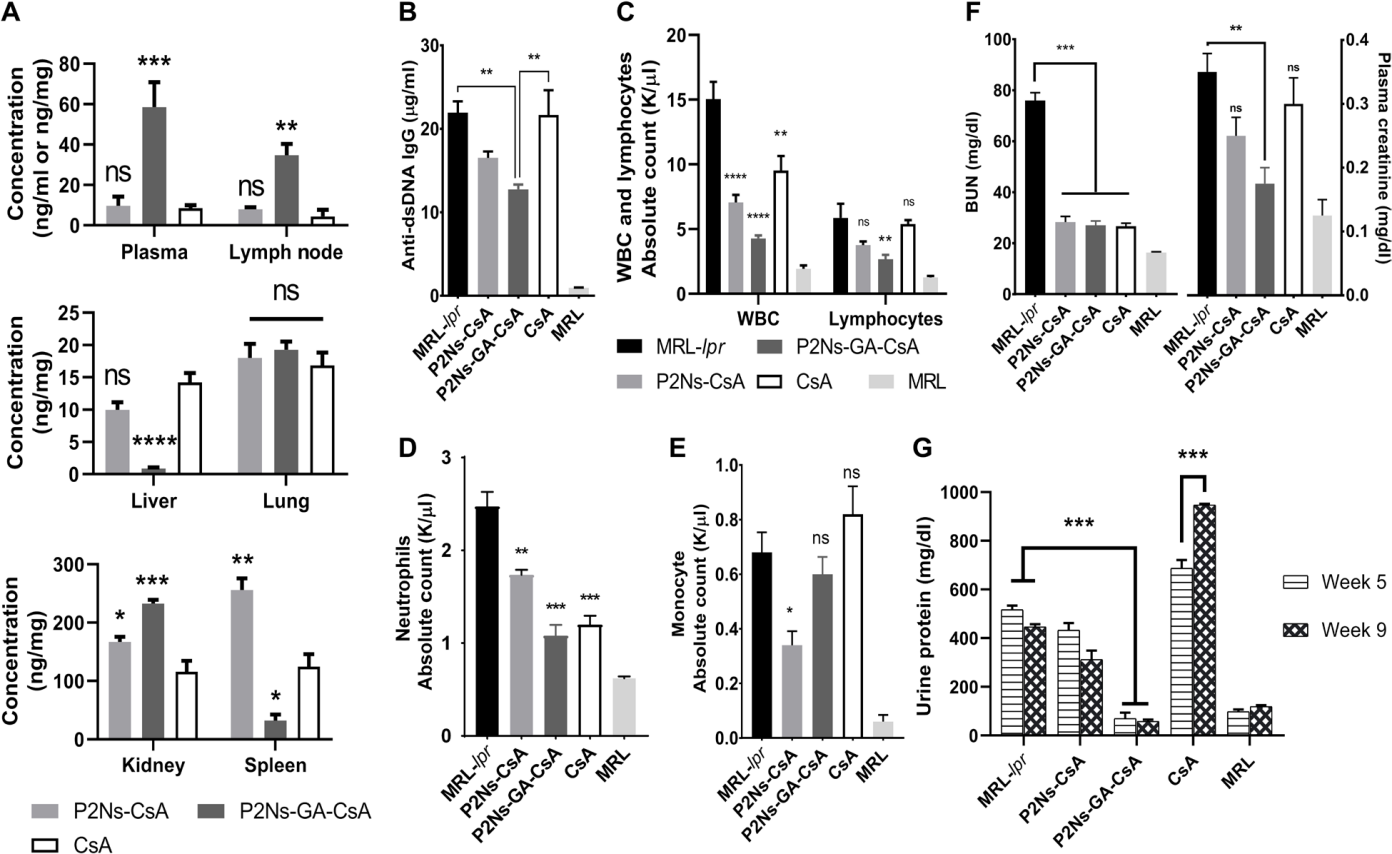
Efficacy of CsA delivery to plasma, lymph nodes, liver, lung, kidney, and spleen, as well as hematological and renal panels. Treatment groups were analyzed after euthanasia for the concentration of CsA delivered to various tissues. The CsA group was dosed about twice as frequently as both P2Ns-CsA and P2Ns-GA-CsA groups (i.e., 5 mg/kg daily versus 5 mg/kg three times per week). (**A**) Significant, 4- to 18-fold increase in CsA plasma and lymph node bioavailability was attained with P2Ns-GA-CsA. Kidney, spleen, liver, and lung CsA concentrations [tissue (ng/mg) or plasma (ng/ml)] were also measured (*n* = 3 to 5 individuals). (**B**) Hematological manifestations of SLE appeared to involve increased plasma anti–double-stranded DNA (dsDNA) immunoglobulin G (IgG), as well as (**C**) white blood cell (WBC), lymphocyte, and (**D**) neutrophil counts, and P2Ns-GA-CsA treatment was able to correct these anomalies except for (**E**) monocyte count (*n* = 4 to 5 individuals). (**F**) Renal functions with respect to blood urea nitrogen (BUN) and plasma creatinine were moderately compromised in MRL-*lpr*, and P2Ns-GA-CsA was capable of reversing these defects (*n* = 3 to 4 individuals). (**G**) Urinary protein elevation was evident in the untreated and P2Ns-CsA– and CsA-treated groups (*n* = 3 to 4 individuals). Data are represented as mean ± SEM. *P < 0.05, **P < 0.01, ***P < 0.001, and ****P < 0.0001. Statistics in (A) were conducted against the CsA group. Statistics in (C) to (E) were conducted against the MRL-*lpr* group. Comparisons were made with one-way ANOVA followed by Tukey posttest.

Tissues collected from the three treatment groups were then assayed for CsA concentrations. Consistent with the higher affinity of P2Ns-GA for lymphocytes, we found that P2Ns-GA-CsA led to significant drug accumulation in the lymph nodes, at a concentration 4- and 18-fold higher than P2Ns-CsA and CsA treatments, respectively (Fig. 3A). P2Ns-GA-CsA treatment also resulted in the highest drug concentration in both the blood plasma and kidneys, with about 14- and 5-fold increase, respectively, over the CsA group, whereas the lung CsA concentrations were similar across all treatment groups (Fig. 3A). The elevated lymphatic disposition of CsA in the P2Ns-GA-CsA group may indicate improved drug transport through the GALT, which drains into many peripheral lymph nodes via efferent mesenteric vessels (*26*). However, because of the interconnected nature of blood and lymphatic vasculature and the high level of plasma CsA in the P2Ns-GA-CsA group, we could not rule out recirculation from blood to lymph as a contributing factor to improved lymph node targeting. In clear juxtaposition with the high plasma and lymphatic bioavailability of CsA in the P2Ns-GA-CsA group, we observed also in this group the lowest CsA levels in the liver and spleen (Fig. 3A). The low CsA concentration in the liver of P2Ns-GA-CsA group was intriguing given this organ’s importance in first-pass metabolism (*23*) and may be consistent with P2Ns-GA-CsA using lymphatic rather than hepatic route of absorption from the small intestine. Low spleen CsA levels in the P2Ns-GA-CsA group, on the other hand, may be explained by a multitude of factors, including the absence of afferent lymphatic vessels to the spleen, changes in drug retention due to different severity of splenomegaly among the treatment groups, changes in the activity of reticuloendothelial system (*27*), and increased drug uptake by nonspleen tissues in the P2Ns-GA-CsA group as a consequence of CD71-mediated active delivery.

The mice were further subjected to blood and urine analyses to detect aberrations in lymphatic and renal function. Untreated MRL-*lpr* mice experienced broad increases in white blood cell, lymphocyte, neutrophil, and monocyte absolute counts, as well as plasma anti–double-stranded DNA (dsDNA) immunoglobulin G (IgG) (a key indicator of lupus) (*2*, *3*), blood urea nitrogen (BUN), creatinine, and urinary protein levels (Fig. 3, B to G). P2Ns-CsA and CsA treatments exhibited varying degree of effectiveness in the reduction of these inflammatory and nephritic markers. In particular, lymphocyte count was not affected by either treatment with statistical significance, even though T cell proliferation should theoretically be down-regulated with CsA (Fig. 3C). While BUN was decreased by all three treatments, CsA and P2Ns-CsA failed to correct plasma creatinine; and considering urine protein levels between fifth and ninth week, CsA also appeared to have adversely affected renal function (Fig. 3F). P2Ns-GA-CsA, meanwhile, substantially normalized all of the above markers with the exception of monocyte count, which remained elevated compared to MRL mice (Fig. 3E).

### Plasma cytokine levels

**Fig. 4:**
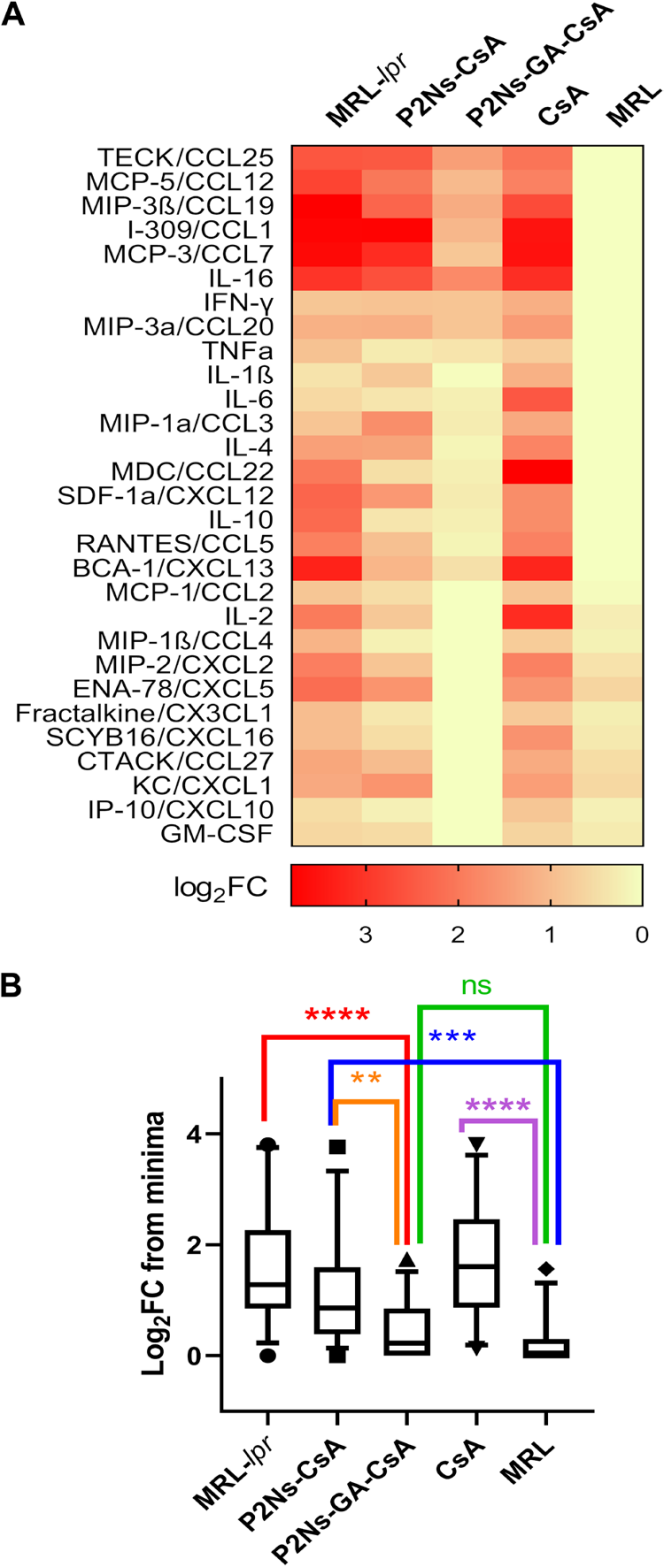
Plasma cytokine/chemokine levels assayed using the multiplex panel. (**A**) SLE-like lymphoproliferation in MRL-*lpr* was accompanied by drastic increases in the concentrations of multiple plasma cytokines/chemokines. Cytokine/chemokine changes were represented as heat map (red, high; yellow, low) and normalized as log-fold increases over the group with the minimum level for a particular cytokine/chemokine. (**B**) Aggregated log-fold increases in plasma cytokines [represented as log_2_FC (fold change) from baseline] was plotted to illustrate the near-global up-regulation of analyzed cytokines/chemokines in the MRL-*lpr* group (leftmost column). Treatments with P2Ns-GA-CsA (middle column) resulted in almost complete normalization of cytokine/chemokine in the plasma. Data are represented as mean ± SEM. **P < 0.01, ***P < 0.001, and ****P < 0.0001; comparisons were made with one-way ANOVA, followed by Tukey’s multiple comparisons test.

A complex array of cytokines and their cognate receptors orchestrate the immune system’s response to and resolution of chronic and acute inflammations. In SLE, control of cytokine levels is oftentimes lost because of breakdowns in feedback mechanisms (*1*, *28*, *29*). Accordingly, we observed drastic increases in a majority of plasma cytokines from the untreated MRL-*lpr* mice compared to negative controls (Fig. 4, A and B). Regulatory and chemotactic cytokines implicated in both innate and adaptive immunity were affected. P2Ns-CsA treatment was marginally effective at normalizing some cytokines, but many remained statistically unchanged (fig. S4). By contrast, owing perhaps to its effective lymphatic targeting, P2Ns-GA-CsA was able to restore almost all of the analyzed cytokines to their baseline levels, affecting not just those directly secreted by lymphocytes (e.g., IL-2, IL-4, IL-6, IL-10, IL-1β, CXCL12, and CXCL13) (*28*, *29*). In contrast to the effectiveness of nanoparticle-based CsA delivery modalities, the CsA treatment not only failed to attenuate cytokine abnormalities but also appeared to trigger statistically significant increases in CCL22, CXCL10, CXCL16, IL-1β, IL-2, and IL-6 (fig. S4). These increases may be caused by the dose insufficiency of the CsA treatment, coupled with slight variations in disease progression among different MRL-*lpr* groups. However, it is also plausible that the CsA group actually underwent more inflammation because of the drug, this being consistent with multiple researches showing that low doses of CsA could counterintuitively augment rather than suppress innate and adaptive immune responses (*30*).

### Histological examination of lymphatic tissues

The systemic lymphoproliferation that we observed at the organ level was further examined with histology. The splenic white pulp, a region of immunological synapse activity and B cell maturation (*31*), was notably expanded in MRL-*lpr*, P2Ns-CsA, and CsA groups (fig. S5A). We continued to observe P2Ns-GA-CsA achieving the most effective treatment outcome as both quantitative measurement of white pulp area and lymphatic cell number and qualitative assessment of Peyer’s patch size demonstrated disease regression (fig. S5, A, C, and D). B (CD20^+^) and T (CD3^+^) cell populations in the spleen were differentially affected by CsA treatments. While CD20^+^ cell numbers were reduced significantly in all three treatment groups, CD3^+^ cell numbers were reduced only with nanoparticle CsA formulations, with those in the P2Ns-GA-CsA group matching the MRL baseline level (fig. S5, B, E, and F).

### Analysis of kidney pathologies

**Fig. 5:**
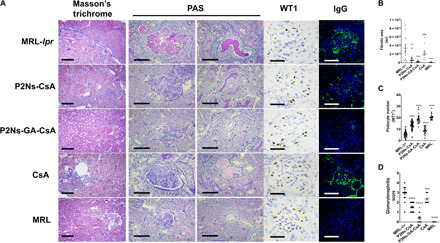
Renal histopathology associated with SLE. (**A**) Representative micrographs showing staining with Masson’s trichrome (first column), periodic acid–Schiff (PAS; second and third columns), anti-WT1 antibody (fourth column), and anti-IgG (fifth column). For both MRL-*lpr* and CsA groups, extensive renal damages were observed, whereas P2Ns-GA-CsA treatment prevented most of those damages (scale bar, 200 μm for Masson’s trichrome staining; scale bars, 50 μm for all other images). (**B**) Graphical representations of fibrosis from Masson’s trichome, (**C**) podocyte count from WT1 staining, and (**D**) glomerulonephritis score from IgG micrographs, for each of the five experimental groups. Representative micrographs are shown; *n* = 15 to 30 images from at least three individuals. Data are represented as mean ± SEM. **P < 0.01, ***P < 0.001, and ****P < 0.0001. Statistics were conducted against the MRL-*lpr* group. Comparisons were made with one-way ANOVA, followed by Tukey’s multiple comparisons test. au, arbitrary units.

Renal dysfunctions from glomerulonephritis are the primary culprit of mortality among SLE-afflicted individuals (*3*). With Masson’s trichrome stain, we observed substantial tubulointerstitial fibrosis in the MRL-*lpr* group, especially surrounding the renal microvasculature (Fig. 5, A and B). Periodic acid–Schiff (PAS) staining revealed additional abnormalities such as thickening of the basement membrane, mesangial expansion, glomerular hypercellularity, and tubular lesions and casts (Fig. 5A). Loss of terminally differentiated podocytes (WT1 staining) (Fig. 5A) and slit diaphragm integrity (nephrin staining) (fig. S6) explained the occurrence of proteinuria and high plasma creatinine in MRL-*lpr*.

Reduction to these pathologies was negligible or borderline in the CsA group, moderate in the P2Ns-CsA group, and almost eliminated in the P2Ns-GA-CsA group (Fig. 5, A and B). Last, immunolabeling of the glomeruli revealed extensive IgG deposits in the mesangial matrix and periglomerular regions of both the CsA and untreated MRL-*lpr* groups but significant reduction in the P2Ns-CsA and P2Ns-GA-CsA groups (Fig. 5A). Using the staining information, we scored multiple kidney sections for glomerulonephritis and found that disease progression was effectively halted by P2Ns-GA-CsA treatment (Fig. 5C).

### Renal recruitment of B and T cells

**Fig. 6:**
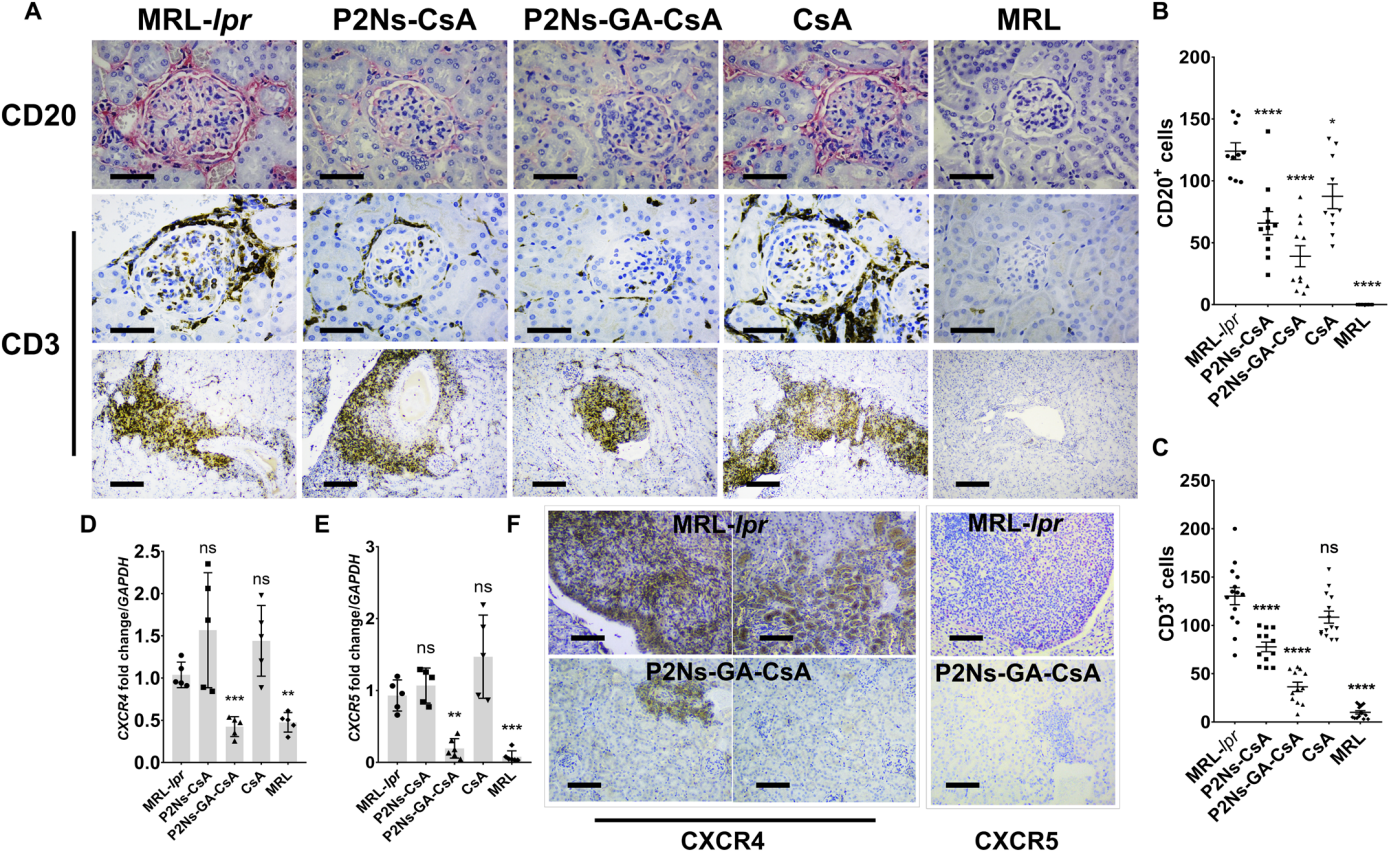
Infiltration of lymphocytes in SLE kidneys. (**A**) Histological images showing that renal damage in the untreated MRL-*lpr* appeared to have a strong correlation with lymphocyte infiltration. Both CD20^+^ and CD3^+^ cells were enriched in the glomeruli (CD20 and CD3) and the interstitial space (CD3) (*n* = 15 to 30 micrographs from at least three individuals; scale bars, 50 μm for first two rows of images; scale bars, 200 μm for third row of images). (**B**) Graphical analyses of CD20^+^ cells and (**C**) CD3^+^ cells in the histological images. (**D**) We observed elevated transcript levels of *CXCR4* and (**E**) *CXCR5* in the untreated MRL-*lpr* mice (*n* = 5 to 6 individuals). (**F**) Renal histology also showed significant up-regulation of CXCR4 (brown) among infiltrating leukocytes and endogenous expression in the proximal tubules. CXCR5^+^ population (red) appeared to decorate the migrating front of infiltrating leukocytes. (scale bars, 200 μm for all images). Representative micrographs are shown. Data are represented as mean ± SEM. *P < 0.05, **P < 0.01, ***P < 0.001, and ****P < 0.0001. Statistics were conducted against the MRL-*lpr* group. Comparisons were made with one-way ANOVA, followed by Tukey’s multiple comparisons test.

Previous studies have shown that renal infiltration of both B and T cells is necessary for the development of lupus nephritis (*1*, *3*). Specific subsets of lymphocytes expressing CXCR4 and CXCR5 also accumulate in the SLE kidney, and antagonism of these receptors has been proposed to alleviate disease severity (*32*, *33*). We have shown that a wide spectrum of cytokines, including CXCL12 and CXCL13 (ligands for CXCR4 and CXCR5, respectively), was up-regulated in the MRL-*lpr* mice, thereby allowing chemotaxis of lymphocytes to the kidneys. With untreated MRL-*lpr* mice, we observed pervasive CD3^+^ and CD20^+^ lymphocyte infiltration throughout the glomeruli, blood vessels, and interstitial space (Fig. 6, A to C). Furthermore, transcripts for *CXCR4* and *CXCR5*, along with the histological signals from these receptors, were markedly higher in the MRL-*lpr* kidney samples, suggesting that an enrichment of CXCR4^+^ and CXCR5^+^ lymphocytes had taken place (Fig. 6, D and E). These histopathological hallmarks were again virtually absent in the P2Ns-GA-CsA group.

## DISCUSSION

Although many immunological pathways involved in SLE pathogenesis have been characterized (*1*–*3*), the discovery of safe and effective drugs (such as antibody-based biologics) targeting these pathways has been challenging and costly (*7*). We believe that an often overlooked, but nonetheless promising approach is to enhance the therapeutic activity of old, well-established drugs with novel delivery methods (*34*). Administration of low-dose CsA (at 3 to 5 mg/kg per day) to patients with SLE has already been confirmed as an effective adjuvant for reduced corticosteroid regimen and concomitant side effects (*35*). Here, we showed that a useful application of nanoparticles could be to transform CsA into a stand-alone therapy for SLE, thereby sparing steroids entirely. A notable advantage of P2Ns-GA-CsA appeared to be 4- to 18-fold improvement in lymph node targeting. As a microenvironment where antigen presentation, affinity maturation, and isotype switching occur, lymph nodes are critical for the pathogenesis of many autoimmune disorders (*36*). For orally delivered drugs, entry into peripheral lymph nodes involves one of two routes: recirculation from blood to lymph or transport through the GALT (*23*). The former route requires initial absorption by the intestinal capillaries and passage through the hepatic portal vein, which inevitably subjects the drug to first-pass metabolism (*23*), a process that can have negative pharmacokinetic consequences. Alternatively, the drug can be brought into lymphatic circulation directly via the GALT, which connects to peripheral lymph nodes through a network of efferent mesenteric vessels (*23*). By achieving better lymphatic bioavailability, drugs that treat inflammatory diseases, metastatic cancers, and infections of lymphopoietic origins should, in theory, acquire greater potency (*37*, *38*).

P2Ns-GA, unlike P2Ns, displayed consistently higher association with CD3^+^, CD20^+^, and CD45^+^ PBMCs, in a manner correlating with CD71 expression. GA-CD71 interaction exhibits high binding affinity according to several in vitro and in silico analyses (*20*, *39*), but this interaction is probably not exclusive among a spectrum of cell surface proteins. Although healthy, ICR mouse lymphocytes were analyzed, there was little evidence suggesting that CD71 expression was reduced in lupus lymphocytes, and on the basis of a publicly available transcriptome database (GSE10325) (*22*), the opposite appeared to be true. In general, mice with lymphoproliferative mutations (such as FAS^lpr/lpr^ in MRL-*lpr* mice) will have much higher numbers of T and B cells in circulation, when compared to normal mice, so it is reasonable to expect more nanoparticle binding to MRL-*lpr* than PBMCs given the greater availability of binding partners. In line with this, we observed increased ex vivo P2Ns-GA binding to CD71^+^/CD3^+^ and CD71^+^/CD20^+^ GALT regions of MRL-*lpr* mice, such as lacteals and Peyer’s patches. The latter, in particular, was known to be involved in the nonspecific uptake of polyester nanoparticles (*40*) and delivering them to lymphatic circulation. Cross sections of Peyer’s patches had generally larger area in untreated MRL-*lpr* mice compared to MRL mice. Whether such disparity further enhances lymphatic delivery of nanoparticles is unknown and certainly warrants further investigation. These observations imply that P2Ns-GA can better exploit the CD71-rich GALT than P2Ns, and potentially hitchhike on lymphocytes migrating between the gut and peripheral lymph nodes.

Although susceptibility to SLE is multifaceted, the failure to effectively suppress autoreactive T cells has been acknowledged as a primary driving force (*2*). In MRL-*lpr* mice, this defect is mimicked by the Fas^lpr/lpr^ mutation, which nullifies a key regulatory checkpoint for the negative selection of immature thymocytes and the clonal deletion of effector T cells by activation-induced cell death (*41*). The uncontrolled lymphoproliferation in the MRL-*lpr* genetic background is manifested in the lymphadenopathy, splenomegaly, and leukocytosis phenotypes (*41*), which we have observed in this study. The expansion of CD3^+^/CD20^+^ populations in lymphoid organs and the ablation of this phenomenon by P2Ns-GA-CsA implied that the formulation was reducing the proliferation of both B and T cells in a Fas-independent manner, but the exact mechanism remained to be elucidated.

Because the administered CsA dose was significantly below the nephrotoxic threshold, we observed few renal irregularities arising from treatments aside from the moderately higher proteinuria in the CsA group. As a popular commercial formulation of CsA, Neoral is a microemulsion that improves upon the bioavailability of its predecessors (*25*). Even so, we observed relatively low plasma CsA levels from the bioequivalent, generic Neoral compared to nanoparticle formulations in our rat pharmacokinetics study (*17*), and the complete lack of efficacy in the present study recapitulated the consensus that CsA must be supported by corticosteroids to treat SLE (*35*). P2Ns-GA-CsA, on the other hand, appeared to have halted SLE progression in virtually every metrics analyzed. Perhaps the most prominent effect was in its normalization of plasma cytokines, several of which, such as CCL5/RANTES, CXCL2/MIP-2, CXCL12/SDF-1α, and CXCL13/BCA-1, were known biomarkers of lupus severity (*32*, *33*, *42*). These positive results may be attributable to much better association of CD3^+^ T cells with P2Ns-GA-CsA compared to P2Ns-CsA, as determined by flow cytometry, which can lead to more CsA-mediated inactivation of these cell types. A significant portion of circulating CD3^+^ T cells in patients with lupus are activated effector cells (*43*) that hyperregulate the systemic inflammatory rheostat by not only secreting substantial amounts of cytokines but also indirectly amplifying cytokine secretion by other lymphoid, myeloid, and epithelial cells (*28*, *29*).

Congruent with high plasma cytokine levels, there was prominent tissue homing of lymphocytes to untreated MRL-*lpr* kidneys, particularly in the periglomerular regions (CD3^+^ and CD20^+^) and tubulointerstitial space (CD3^+^). These events were accompanied by diffused in situ IgG depositions, basement membrane thickening, mesangial expansion, tubular casts, fibrotic lesions, loss of podocytes, and disruption of slit diaphragm, all of which indicative of moderate to severe nephritis (*3*). We also observed elevated signals of CXCR4 and CXCR5 in MRL-*lpr* kidneys with quantitative polymerase chain reaction (PCR) and histology, implying that there could be recruitment of highly pro-inflammatory, CXCR4-expressing B cells (*32*) and CXCR5-expressing double-negative T cells (*33*) to the renal parenchyma. P2Ns-GA-CsA treatment returned much of the SLE-associated renal histopathology, along with protein filtration, BUN, and plasma creatinine to their normal states. The near total inhibition of immune-related tissue damage by P2Ns-GA-CsA suggested that the treatment may be targeting a signaling component fundamental to the etiology of lupus. Because the reconfiguration of T cell receptor (TCR) by FcRγ/Syk, increased Ca^++^ influx, and calcineurin hyperactivation (*43*) were known defects of SLE T cells, intervention with CsA (at the sufficient dosage and acting on the appropriate lymphoid tissue) may drastically attenuate autoreactive T cell activation, thereby leading to the positive outcomes observed in this study.

Previous research on CsA has posited a direct relationship between the inhibition of calcineurin/NFAT signaling and the development of autoimmunity (*44*, *45*). Such seemingly paradoxical observation arises from CsA’s disruption of NFAT-induced expression of *Fas*, which serves to limit maximum TCR response by mediating the negative selection of autoreactive thymocytes and the clonal deletion of effector T cells (*41*). We could not investigate changes in Fas activity among our treatment groups primarily because the MRL-*lpr* mice were genetically defective in this receptor. Nevertheless, because many of the CsA-autoimmunity studies were performed using neonatal, irradiated, or otherwise immunocompromised animals, their relevance on preexisting autoimmune conditions such as SLE seemed questionable. CsA had been investigated as a possible therapy for atopic dermatitis, psoriasis, uveitis, and rheumatoid arthritis (*46*), making it unlikely that the drug will augment autoimmunity in situations where the basal immune response is already high. Future experiments, however, will seek to elucidate the mechanisms behind P2Ns-GA-CsA’s modulation of autoimmunity, particularly with respect to T cell subsets. As cytotoxic, regulatory, T helper 1 (T_H_1)/2/17, double-negative, and follicular helper T cells each have important and sometimes diametrically opposite roles to play on the progression of SLE (*43*), it is beneficial to understand how the administration of P2Ns-GA-CsA may affect their long-term balance.

There has been much debate in the literature surrounding the question of particle size cutoff of nonconventional (nanoparticle) formulations for drug uptake following oral dosing. Despite the fact that even though there is no agreement on the cutoff for particle size for optimal oral absorption, it is a relative phenomenon whereby smaller is generally believed to be better. Recent studies suggest that smaller (0.5 μm) vehicles lead to greater uptake compared to larger (5 μm) vehicles. The polymer systems will invariably have polydispersity coming from polymers/emulsion-based preparations, and this, to some extent, can influence the pharmacokinetic profile, with a probability of affecting pharmacodynamics; however, such experimental reports in literature are scarce. To this extent, our earlier reports suggest that particle size (nanoscale versus microscale) showed that nanoscale particles (~300 nm) were moderately better at the oral delivery of CsA compared to microscale particles (~1.2 μm) (*47*). Last, it remains to be explored the full extent of P2Ns-GA-CsA efficacy in murine SLE models, including, but not limited to, survival analysis, dose-dependent response, the effect of particle size, induction of drug treatment at different stages of SLE, and the presence of side effects from chronic immunosuppressant use. These studies will allow a more comprehensive insight into the pharmacological strengths and weaknesses of P2Ns-GA-CsA and establish its translational potential for human SLE treatment.

## MATERIALS AND METHODS

### Mice and reagents

MRL/MpJ-Fas^lpr^/J (MRL-*lpr*) and MRL/MpJ (MRL) mice were obtained from the Jackson laboratory (Bar Harbor, ME). Blood from ICR mice was made available through the Texas A&M Comparative Medicine Program (CMP) tissue share, which was accredited by the Association for the Assessment and Accreditation of Laboratory Animal Care, International. All reagents were analytic grade, unless mentioned otherwise, and were purchased from Fisher Scientific (Hampton, NH). GA was purchased from BroadPharm (San Diego, CA). CsA to be used for nanoparticle formulations was purchased from Alfa Aesar (Haverhill, MA). Generic Neoral or cyclosporine USP (US Pharmacopeia) modified (100-mg capsule, Teva Pharmaceutical Industries, Petah Tikva, Israel) was purchased through CMP. Lymphoprep and SepMate tubes were purchased from STEMCELL Technologies (Vancouver, Canada). Hydrophobic LabSand for urine collection was purchased from Braintree Scientific (Braintree, MA). Immunohistochemistry and immunofluorescence antibodies for CD20 (polyclonal) and IgG (polyclonal, Alexa Fluor 488) were purchased from Thermo Fisher Scientific (Waltham, MA); antibodies for WT1 (D8175), CXCR4 (D4Z7W), and CXCR5 (D6L3C) were purchased from Cell Signaling Technology (Danvers, MA); antibody for CD3 (polyclonal) was purchased from Agilent (Santa Clara, CA); and antibody for nephrin (polyclonal) was purchased from R&D Systems (Minneapolis, MN). Fluorescent antibodies for CD71 (RI7217), CD20 (SA275A11), CD3 (17A2), and CD45 (30-F11) were purchased from BioLegend (San Diego, CA). DNA primers were ordered at standard purity and without modification from Integrated DNA Technologies (Coralville, IA).

### Synthesis of polymer

Prepolymer containing l-lactide and PEG 400 was synthesized using a two-step polycondensation method. The first step was a ring-opening reaction, which provided the required backbone for the polymer. The polymeric chain was further drawn-out using a chain extension compound, cyclohexanetetracarboxylic dianhydride (HCDA), in the second step of the reaction. The final polymer obtained, called Precision Polymer (P2s), had the appearance of a white powder with six to eight free carboxylic acids along the polyester backbone.

^1^H nuclear magnetic resonance (NMR) spectra of the prepolymer (PLA-PEG-PLA) confirmed peaks at 1.55 to 1.58 parts per million (ppm) and 5.11 to 5.15 ppm, designated to PLA methyl and methylene protons, respectively, and at 3.60 to 3.62 ppm, designated to PEG methylene. The peaks at 3.65 and 4.20 to 4.30 ppm were due to PEG methylene linkage to the carbonyl ester ends of PLA. P2s obtained with chain extension revealed the presence of HCDA using ^13^C NMR spectroscopy, in addition to the aforementioned PLA and PEG peaks.

### Conjugating GA with P2s

To achieve the extra functionality and better absorption of the nanoparticles, P2s was decorated with GA using the linker ethylene diamine and EDC chemistry. All the free carboxylic acids on P2s were coupled via amide bonding with ethylene diamine through one of the latter’s free amines, and then, GA was conjugated to the polymer using the remaining free amine on the ethylene diamine linker. The GA-modified polymer, called P2s-GA, was confirmed using ^1^H NMR, in which peak at 8 ppm corresponded to the presence of amide bond, while doublets at 5.5 and 7.5 ppm are the characteristic peaks of GA. Fourier transform infrared spectroscopy further confirms correct polymer synthesis with C═O stretching at 1640 to 1690 cm^−1^ and N─H bending at 1550 to 1640 cm^−1^.

### CsA and fluorescent P2Ns/P2Ns-GA

CsA nanoformulations (P2Ns-CsA or P2Ns-GA-CsA) were prepared by a scaled-up version of the single emulsification process described previously by our group (*17*). Because GA was a high–molecular weight molecule, there would be significant weight loss of the polymer during preparation of P2s-GA. To compensate for that loss, equivalent amount of P2s was added to P2s-GA during the preparation of nanoformulation (P2Ns-GA-CsA). The process consisted of two steps: emulsification and evaporation. In brief, (i) organic phase containing 500 mg of polymer (P2s or P2s-GA) was initially stirred for 10 min in 5 ml of dichloromethane, followed by adding 15 ml of ethyl acetate. Separately, 20% (w/w) CsA was dissolved in 5 ml of ethyl acetate. After 1 hour, both polymer and drug were mixed and stirred for further 30 min. (ii) Aqueous phase containing 500 mg of polyvinyl alcohol in 50 ml of water was prepared and used to emulsify the polymer/drug-containing organic phase with homogenization at 15,000 rpm for 30 min. This emulsion was then added to 200 ml of water to facilitate the diffusion of organic solvent, which was subsequently evaporated over 3 to 4 hours. The resultant suspension was then centrifuged at 15,000*g* for 30 min at 4°C. The pellet was suspended in 25 ml of 5% (w/v) sucrose solution and frozen overnight at −80°C. Freeze-drying of this suspension was carried out using a benchtop freeze dryer (FreeZone Triad, Labconco, Kansas City, MO) at −55°C for 54 hours, followed by heating at 20°C for 20 hours under vacuum (0.008 mbar). The freeze-dried product was crimp sealed and stored at 4°C until further use. The resulting nanoparticle preparations contained approximately 0.22 mg of CsA per 1 mg of polymer, identified according to previously reported methods (*17*). When compared to most of the literature that usually report 50-mg lots, this batch size was about 10 times higher than such preparations. The accompanying fig. S1 depicts the representative data from polymer synthesis and nanoparticle formulations.

Fluorescent nanoparticles of P2Ns and P2Ns-GA were prepared as described by our group (*17*). In brief, the amine-reactive *N*-hydroxysuccinimide–fluorescein was conjugated to P2s via the ethylene diamine linker to form P2s-FL (fluorescent) prepolymers. Thereafter, P2s-FL was mixed with P2s or P2s-GA prepolymers at 1:24 (w/w) ratio in dichloromethane and ethyl acetate before emulsification and evaporation in aqueous solvent containing 1% didodecyl dimethylammonium bromide (DMAB). The nanoparticle pellets were recovered after centrifugation, suspended in 5% trehalose dihydrate solution, and freeze-dried.

### PBMC isolation

PBMCs were isolated from the whole blood of ICR mice. Blood samples collected from approximately 50 16-week-old female mice were pooled. Phosphate-buffered saline (PBS) + 2% fetal bovine serum (FBS) was used to dilute the blood at 1:1 ratio, and then, the mixture was slowly added to 4.5 ml of Lymphoprep density gradient medium in a SepMate tube (both from STEMCELL Technologies). The tubes were centrifuged at 1200*g* for 20 min at room temperature to segregate the PBMCs, which would be enriched in the top layer. The PBMCs were decanted into new tubes, washed two times with cold PBS + 2% FBS, and suspended in freezing medium (Bambanker). The cells were counted by hemocytometer and stored at −80°C.

### In vitro nanoparticle binding assay

From the same frozen stock, each sample consisting of approximately 2 × 10^5^ PBMCs was incubated with 50 μg of FITC-labeled P2Ns or P2Ns-GA for 60 min at room temperature. PE/Cy7-conjugated antibody against CD71 (0.25 μg/ml per sample), along with APC-conjugated antibodies against either CD3 (0.5 μg/ml per sample), CD20 (0.5 μg/ml per sample), or CD45 (0.25 μg per sample), was then added to the PBMCs and incubated for 15 to 20 min on ice. All antibodies were diluted in PBS containing 3% goat serum. After incubation, the PBMCs were washed three times in PBS + 2% FBS, and 5 μl of 7-amino-actinomycin D (7AAD) (Invitrogen, Carlsbad, CA) was added to discriminate between live and dead cells. Flow cytometry was performed with MoFlo Astrios EQ (Beckman Coulter, Brea, CA), and data analyses were carried out with FlowJo version 10 (Becton Dickinson, Franklin Lakes, NJ). Following flow cytometry, PBMCs were fixed in 4% formalin and mounted onto glass slides with a mounting medium containing DAPI (4′,6-diamidino-2-phenylindole; Vectashield with DAPI, Vector Laboratories, Burlingame, CA). Fluorescent images of individual PBMCs were captured at 40× with confocal microscopy (Zeiss LSM 780, Oberkochen, Germany).

### Ex vivo intestinal binding assay

Unstained, formalin-fixed and paraffin-embedded (FFPE) tissue sections (5-μm thickness, on glass slides) were obtained from 17-week-old, untreated MRL-*lpr* mice. Following deparaffinization and hydration, antigen retrieval was undertaken by boiling in sodium citrate buffer (0.01 M, pH 6.0) for 5 min and three times. Fifty micrograms of either FITC-labeled P2Ns or P2Ns-GA was added onto each sample slide and incubated for 60 min at 37°C. After incubation, the slides were rinsed several times with PBS to remove unbound nanoparticles and stained with PE/Cy7-conjugated anti-CD71 antibody (0.25 μg/ml) and either APC-conjugated anti-CD3 or anti-CD20 antibody (0.5 μg/ml) for 2 hours at room temperature. All antibodies were diluted in PBS containing 3% goat serum. The sections were mounted using Vectashield plus DAPI to visualize nuclei and imaged with LSM 780 confocal laser scanning microscope (Zeiss). Microscopy images were processed using ZEN 2 Black Edition image processing software. Colocalization analysis was performed with Coloc2 plugin in ImageJ software to compute Manders’ overlap coefficients. Images were at taken at ×20 (small intestines) or ×40 (PBMCs) magnification with image dimension of 212 μm by 212 μm or 106 μm by 106 μm, respectively.

### Efficacy study in SLE model mice

MRL-*lpr* and MRL mice, aged 7 weeks, were acclimatized for 1 week before study. The mice were then divided into five groups with the following sample size and composition: groups 1 to 4 (designated MRL-*lpr*, P2Ns-CsA, P2Ns-GA-CsA, and CsA, respectively)—five female MRL-*lpr* mice; and group 5 (designated MRL)—three female and three male MRL mice. The animals were housed at five per cage (or three per cage and gender-segregated for group 5). Starting from the eighth week of age, CsA formulations were administered via oral gavage to each mouse in groups 2 to 4, whereas groups 1 and 5 had no intervention. Groups 2 and 3 received P2Ns-CsA or P2Ns-GA-CsA formulations, respectively, at 5 mg/kg (CsA equivalent) and with three doses per week (i.e., Monday, Wednesday, and Friday). Group 4 received generic Neoral (CsA) at 5 mg/kg and with daily dosing. Mice were weighed at the beginning of each week to adjust dosage. At treatment weeks 5 and 9 (or weeks 12 and 16 of age, respectively), using a clean cage with approximately 0.5 kg of hydrophobic sand (LabSand), individual mouse was placed therein, and urine droplets on LabSand were collected. The lymph node weight at the 17th week of age (at sacrifice) was measured using digital balance. Thereafter, all mice were sacrificed by CO_2_ asphyxiation and cervical dislocation. Relevant tissues (i.e., kidneys, spleen, thymus, cervical, and axillary lymph nodes) were immediately isolated, washed in ice-cold saline, and weighed. Tissues were split between 10% formalin fixation or flash-frozen in liquid N_2_, followed by −80°C storage. To compute hypertrophy, the major dimensions of spleen and thymus were measured while freshly dissected. Blood from cardiac puncture was collected in heparinized tubes and split into two fractions; one of which was spun down at 3000 rpm for 20 min, after which plasma was decanted and stored at −80°C; the other whole-blood fraction was immediately sent for hematological analyses at 4°C. All animal experimentations were approved by the Texas A&M University Institutional Animal Care and Use Committee and assigned as protocol no. 2018-0062.

### Urine and blood analyses

Approximately 300 to 500 μl of whole blood per sample per mouse were sent to the Texas A&M Veterinary Medical Diagnostic Laboratory (TVMDL), which provided service for blood panel, BUN, and creatinine analyses. Urine samples procured at treatment weeks 5 and 9 with hydrophobic LabSand were also sent to TVMDL to quantify protein content.

### Histopathology

After at least 2 weeks of fixation in 10% formalin, tissues were stored in 70% ethanol and then sent for paraffin sectioning. Both sstained and unstained FFPE tissue sections (5-μm thickness) were prepared by the Texas A&M College of Veterinary Medicine (CVM) Histology Laboratory. Staining with hematoxylin and eosin, PAS, PAS/Alcian blue, or Masson’s trichrome reagents was applied to hydrated sections using standard protocols. Immunohistochemistry was carried out using primary antibodies against mouse CD3 (1:300), CD20 (1:500), CXCR4 (1:300), CXCR5 (1:300), and WT1 (1:300). Secondary antibodies conjugated to AP (alkaline phosphatase) were used for CD20 and CXCR5, whereas those conjugated to horseradish peroxidase were used for CD3, CXCR4, and WT1. Chromogenic signals were developed using commercial kits for DAB (ImmunoCruz Rabbit ABC Staining System, Santa Cruz, Dallas, TX) and Warp Red (MACH 4 Universal AP Polymer Kit, Biocare, Pacheco, CA). The sections were counterstained with hematoxylin before dehydration and mounting. Direct immunofluorescence was carried out using fluorescent antibody against IgG (1:500). Primary antibody against nephrin (1:1000) was followed by fluorescent secondary antibody (NL557) to assay for slit diaphragm. The sections were mounted with a medium containing DAPI (Vectashield with DAPI, Vector Laboratories). Stitched ultraresolution images were captured using Cytation 5 (BioTek, Winooski, VT) at ×4 magnification, and single images were captured using confocal microscopy (Zeiss LSM 780) at ×40 magnification, or with a bright-field microscope at ×4 and ×40 magnification (Accu-Scope, Commack, NY). At fifteen to 30 representative images taken from different mice within the same group were subjected to subsequent analyses. The built-in analyze feature of ImageJ was used to determine CD3^+^/CD20^+^ cell number, white pulp area, fibrotic area (blue color in Masson’s trichrome), podocyte number, and nephrin density. Briefly, full-resolution images were loaded into ImageJ, and regions of interest (e.g., those with CD3/CD20, white pulp, and WT1 containing pixels) were selected under color threshold. Threshold regions were then measured by particle analysis to determine their number and average and total size. Monochromatic images from the anti-nephrin antibody fluorescent channel were directly subjected to measurement analysis to determine the integrated density of signals. Assessment of glomerulonephritis scores was based on the standard set forth by the International Society of Nephrology and International Pathology Society Classification of lupus nephritis. Lymph node cell count was computed by Cytation 5 (BioTek) using the built-in cell count algorithm.

### Immunoassay of CsA, anti-dsDNA IgG, and chemokines/cytokines

For CsA determination, portions of the cervical lymph node, kidney, and spleen were excised from the same anatomical positions of the whole frozen tissue and, after weighing, were immersed in PBS at 1:9 (w/v) ratio. Tissue samples were then homogenized with probe sonicator (Qsonica, Cole-Parmer, Vernon Hills, IL) at 40% amplitude for 20 to 40 s. Plasma samples were diluted 1:5 with PBS. CsA concentrations from tissue homogenates and diluted plasma samples were determined with the Mouse Cyclosporine A ELISA Kit (MBS720488, MyBioSource, San Diego, CA). For antinuclear antibody determination, plasma samples diluted 1:800 with PBS were used as inputs to Mouse Anti-dsDNA IgG Antibody Assay Kit (no. 3031, Chondrex, Redmond, WA). Signals from both CsA and anti-dsDNA IgG assays were detected using a microplate reader (TECAN Infinite M200, Morrisville, NC). Chemokine/cytokine concentrations were assayed with Bio-Plex Pro Mouse Chemokine Panel 33-Plex (Bio-Rad, Hercules, CA) using plasma samples diluted at 1:5 with the manufacturer-provided diluent solution. Signals from the multiplex panel were detected using the Bio-Plex 200 system (Bio-Rad). All assays were performed according to the manufacturer’s instructions. Heat map was generated by first calculating the log_2_FC (fold change) of each chemokine/cytokine’s mean concentration in each group compared against the group with the lowest mean concentration observed for that particular chemokine/cytokine. All log_2_FC values were positive numbers with zero indicating no change, and all other values indicating increase from baseline. The array containing log_2_FC of all cytokines/chemokines from all groups was imported into GraphPad Prism version 8.01 to create the dual-color heat map.

### Real-time PCR

Mice kidneys were cut at the midpoint, along the transverse plane, to obtain slices of approximately 5 mg. These were then homogenized in E.Z.N.A. Total RNA Kit I (Omega Bio-Tek, Norcross, GA) using a handheld tissue homogenizer. RNA was extracted by following the manufacturer’s instructions and quantified at 260 nm with a microplate reader (TECAN Infinite M200). Genomic DNA digestion and reverse transcription were carried out with iScript gDNA Clear cDNA Synthesis Kit (Bio-Rad) using 1 μg of RNA input. The CFX96 Touch Real-Time PCR Detection System (Bio-Rad), along with the iTaq Universal SYBR Green Supermix (Bio-Rad), was used to amplify target transcripts with primers: 5′-GAAGTGGGGTCTGGAGACTAT-3′ and 5′-TTGCCGACTATGCCAGTCAAG-3′ for *CXCR4*, 5′-ATGAACTACCCACTAACCCTGG-3′ and 5′-TGTAGGGGAATCTCCGTGCT-3′ for *CXCR5*, and 5′-AGGTCGGTGTGAACGGATTTG-3′ and 5′-TGTAGACCATGTAGTTGAGGTCA for glyceraldehyde-3-phosphate dehydrogenase (*GAPDH*). Δ*C*_t_ values were calculated with *GAPDH* normalization, and fold change of the gene of interest was expressed as 2^−ΔΔ*C*t^.

### Statistical analysis

Statistical analysis was performed using GraphPad Prism version 8.01. For PBMC in vitro nanoparticle binding MFI, % events, and urine protein, the unpaired Student’s *t* test was used to compare the groups. For all other experiments, one-way analysis of variance, followed by Tukey’s multiple comparisons test, was performed. Significant differences were considered when *P* < 0.05.

## Supplementary Material

abb3900_Movie_S5.avi

abb3900_Movie_S3.avi

abb3900_Movie_S4.avi

abb3900_Movie_S1.avi

abb3900_SM.pdf

abb3900_Movie_S2.avi

## SUPPLEMENTARY MATERIALS

Supplementary material for this article is available at http://advances.sciencemag.org/cgi/content/full/6/24/eabb3900/DC1

## Appendix

View/request a protocol for this paper from *Bio-protocol*.

## References

[1] Tsokos G. C., Lo M. S., Costa Reis P., Sullivan K. E., New insights into the immunopathogenesis of systemic lupus erythematosus. Nat. Rev. Rheumatol. 12, 716–730 (2016). 27872476 10.1038/nrrheum.2016.186

[2] Moulton V. R., Tsokos G. C., T cell signaling abnormalities contribute to aberrant immune cell function and autoimmunity. J. Clin. Invest. 125, 2220–2227 (2015). 25961450 10.1172/JCI78087 PMC4497749

[3] Mohan C., Putterman C., Genetics and pathogenesis of systemic lupus erythematosus and lupus nephritis. Nat. Rev. Nephrol. 11, 329–341 (2015). 25825084 10.1038/nrneph.2015.33

[4] Xiong W., Lahita R. G., Pragmatic approaches to therapy for systemic lupus erythematosus. Nat. Rev. Rheumatol. 10, 97–107 (2014). 24166241 10.1038/nrrheum.2013.157

[5] Ruiz-Irastorza G., Danza A., Khamashta M., Glucocorticoid use and abuse in SLE. Rheumatology 51, 1145–1153 (2012). 22271756 10.1093/rheumatology/ker410

[6] Campar A., Farinha F., Vasconcelos C., Refractory disease in systemic lupus erythematosus. Autoimmun. Rev. 10, 685–692 (2011). 21600313 10.1016/j.autrev.2011.04.027

[7] Murphy G., Isenberg D. A., New therapies for systemic lupus erythematosus—Past imperfect, future tense. Nat. Rev. Rheumatol. 15, 403–412 (2019). 31165780 10.1038/s41584-019-0235-5

[8] Holdsworth S. R., Gan P. Y., Kitching A. R., Biologics for the treatment of autoimmune renal diseases. Nat. Rev. Nephrol. 12, 217–231 (2016). 26949177 10.1038/nrneph.2016.18

[9] Naesens M., Kuypers D. R., Sarwal M., Calcineurin inhibitor nephrotoxicity. Clin. J. Am. Soc. Nephrol. 4, 481–508 (2009). 19218475 10.2215/CJN.04800908

[10] Matsuda S., Koyasu S., Mechanisms of action of cyclosporine. Immunopharmacology 47, 119–125 (2000). 10878286 10.1016/s0162-3109(00)00192-2

[11] Thomson A. W., The effects of cyclosporin A on non-T cell components of the immune system. J. Autoimmun. 5 ( Suppl A), 167–176 (1992). 1503609 10.1016/0896-8411(92)90031-k

[12] Ina K., Kusugami K., Shimada M., Tsuzuki T., Nishio Y., Binion D. G., Imada A., Ando T., Suppressive effects of cyclosporine A on neutrophils and T cells may be related to therapeutic benefits in patients with steroid-resistant ulcerative colitis. Inflamm. Bowel Dis. 8, 1–9 (2002). 11837932 10.1097/00054725-200201000-00001

[13] Losa J. G., Mateos F. R., Jiménez A. L., Pérez J. A., Action of cyclosporin A on mononuclear phagocytes. J. Investig. Allergol. Clin. Immunol. 6, 222–231 (1996). 8844498

[14] Dammacco F., Della Casa O., Ferraccioli G., Racanelli V., Casatta L., Bartoli E., Cyclosporine-A plus steroids versus steroids alone in the 12-month treatment of systemic lupus erythematosus. Int. J. Clin. Lab. Res. 30, 67–73 (2000). 11043499 10.1007/s005990070017

[15] Fava A., Petri M., Systemic lupus erythematosus: Diagnosis and clinical management. J. Autoimmun. 96, 1–13 (2019). 30448290 10.1016/j.jaut.2018.11.001 PMC6310637

[16] Saini P., Ganugula R., Arora M., Kumar M. N. V. R., The next generation non-competitive active polyester nanosystems for transferrin receptor-mediated peroral transport utilizing gambogic acid as a ligand. Sci. Rep. 6, 29501 (2016). 27388994 10.1038/srep29501 PMC4937428

[17] Ganugula R., Arora M., Saini P., Guada M., Kumar M. N. V. R., Next generation precision-polyesters enabling optimization of ligand–receptor stoichiometry for modular drug delivery. J. Am. Chem. Soc. 139, 7203–7216 (2017). 28395139 10.1021/jacs.6b13231

[18] Banerjee D., Flanagan P. R., Cluett J., Valberg L. S., Transferrin receptors in the human gastrointestinal tract. Relationship to body iron stores. Gastroenterology 91, 861–869 (1986). 3017805 10.1016/0016-5085(86)90687-6

[19] Ned R. M., Swat W., Andrews N. C., Transferrin receptor 1 is differentially required in lymphocyte development. Blood 102, 3711–3718 (2003). 12881306 10.1182/blood-2003-04-1086

[20] Arora M., Ganugula R., Kumar N., Kaur G., Pellois J.-P., Garg P., Kumar M. N. V. R., Next-generation noncompetitive nanosystems based on gambogic acid: In silico identification of transferrin receptor binding sites, regulatory shelf stability, and their preliminary safety in healthy rodents. ACS Appl. Bio. Mater. 2, 3540–3550 (2019). 10.1021/acsabm.9b00419 PMC6705617 31440745

[21] Lemay S., Mao C., Singh A. K., Cytokine gene expression in the MRL/lpr model of lupus nephritis. Kidney Int. 50, 85–93 (1996). 8807576 10.1038/ki.1996.290

[22] Becker A. M., Dao K. H., Han B. K., Kornu R., Lakhanpal S., Mobley A. B., Li Q.-Z., Lian Y., Wu T., Reimold A. M., Olsen N. J., Karp D. R., Chowdhury F. Z., Farrar J. D., Satterthwaite A. B., Mohan C., Lipsky P. E., Wakeland E. K., Davis L. S., SLE peripheral blood B cell, T cell and myeloid cell transcriptomes display unique profiles and each subset contributes to the interferon signature. PLOS ONE 8, e67003 (2013). 23826184 10.1371/journal.pone.0067003 PMC3691135

[23] O’Driscoll C. M., Lipid-based formulations for intestinal lymphatic delivery. Eur. J. Pharm. Sci. 15, 405–415 (2002). 12036717 10.1016/s0928-0987(02)00051-9

[24] Jung C., Hugot J. P., Barreau F., Peyer’s patches: The immune sensors of the intestine. Int. J. Inflam. 2010, 823710 (2010). 21188221 10.4061/2010/823710 PMC3004000

[25] Ankola D., Wadsworth R., Kumar M. N. V. R., Nanoparticulate delivery can improve peroral bioavailability of cyclosporine and match Neoral Cmax sparing the kidney from damage. J. Biomed. Nanotechnol. 7, 300–307 (2011). 21702368 10.1166/jbn.2011.1278

[26] Koboziev I., Karlsson F., Grisham M. B., Gut-associated lymphoid tissue, T cell trafficking, and chronic intestinal inflammation. Ann. N. Y. Acad. Sci. 1207 ( Suppl 1), E86–E93 (2010). 20961311 10.1111/j.1749-6632.2010.05711.x PMC3075575

[27] Cataldi M., Vigliotti C., Mosca T., Cammarota M., Capone D., Emerging role of the spleen in the pharmacokinetics of monoclonal antibodies, nanoparticles and exosomes. Int. J. Mol. Sci. 18, E1249 (2017). 28604595 10.3390/ijms18061249 PMC5486072

[28] Raphael I., Nalawade S., Eagar T. N., Forsthuber T. G., T cell subsets and their signature cytokines in autoimmune and inflammatory diseases. Cytokine 74, 5–17 (2015). 25458968 10.1016/j.cyto.2014.09.011 PMC4416069

[29] Vazquez M. I., Catalan-Dibene J., Zlotnik A., B cells responses and cytokine production are regulated by their immune microenvironment. Cytokine 74, 318–326 (2015). 25742773 10.1016/j.cyto.2015.02.007 PMC4475485

[30] Flores C., Fouquet G., Moura I. C., Maciel T. T., Hermine O., Lessons to learn from low-dose cyclosporin-A: A new approach for unexpected clinical applications. Front. Immunol. 10, 588 (2019). 30984176 10.3389/fimmu.2019.00588 PMC6447662

[31] Lewis S. M., Williams A., Eisenbarth S. C., Structure and function of the immune system in the spleen. Sci. Immunol. 4, eaau6085 (2019). 30824527 10.1126/sciimmunol.aau6085 PMC6495537

[32] Zhao L. D., Liang D., Wu X. N., Li Y., Niu J. W., Zhou C., Wang L., Chen H., Zheng W. J., Fei Y. Y., Tang F. L., Li Y. Z., Zhang F. C., He W., Cao X. T., Zhang X., Contribution and underlying mechanisms of CXCR4 overexpression in patients with systemic lupus erythematosus. Cell. Mol. Immunol. 14, 842–849 (2017). 27665947 10.1038/cmi.2016.47 PMC5649106

[33] Wiener A., Schippers A., Wagner N., Tacke F., Ostendorf T., Honke N., Tenbrock K., Ohl K., CXCR5 is critically involved in progression of lupus through regulation of B cell and double-negative T cell trafficking. Clin. Exp. Immunol. 185, 22–32 (2016). 26990531 10.1111/cei.12791 PMC4908294

[34] Soppimath K. S., Aminabhavi T. M., Kulkarni A. R., Rudzinski W. E., Biodegradable polymeric nanoparticles as drug delivery devices. J. Control. Release 70, 1–20 (2001). 11166403 10.1016/s0168-3659(00)00339-4

[35] Moroni G., Doria A., Ponticelli C., Cyclosporine (CsA) in lupus nephritis: Assessing the evidence. Nephrol. Dial. Transplant. 24, 15–20 (2009). 18852191 10.1093/ndt/gfn565

[36] Alitalo K., The lymphatic vasculature in disease. Nat. Med. 17, 1371–1380 (2011). 22064427 10.1038/nm.2545

[37] Maisel K., Sasso M. S., Potin L., Swartz M. A., Exploiting lymphatic vessels for immunomodulation: Rationale, opportunities, and challenges. Adv. Drug Deliv. Rev. 114, 43–59 (2017). 28694027 10.1016/j.addr.2017.07.005 PMC6026542

[38] Cao S., Slack S. D., Levy C. N., Hughes S. M., Jiang Y., Yogodzinski C., Roychoudhury P., Jerome K. R., Schiffer J. T., Hladik F., Woodrow K. A., Hybrid nanocarriers incorporating mechanistically distinct drugs for lymphatic CD4+ T cell activation and HIV-1 latency reversal. Sci. Adv. 5, eaav6322 (2019). 30944862 10.1126/sciadv.aav6322 PMC6436934

[39] Kasibhatla S., Jessen K. A., Maliartchouk S., Wang J. Y., English N. M., Drewe J., Qiu L., Archer S. P., Ponce A. E., Sirisoma N., Jiang S., Zhang H. Z., Gehlsen K. R., Cai S. X., Green D. R., Tseng B., A role for transferrin receptor in triggering apoptosis when targeted with gambogic acid. Proc. Natl. Acad. Sci. U.S.A. 102, 12095–12100 (2005). 16103367 10.1073/pnas.0406731102 PMC1189297

[40] Shakweh M., Ponchel G., Fattal E., Particle uptake by Peyer’s patches: A pathway for drug and vaccine delivery. Expert Opin. Drug Deliv. 1, 141–163 (2004). 16296726 10.1517/17425247.1.1.141

[41] Nagata S., Mutations in the Fas antigen gene in *lpr* mice. Semin. Immunol. 6, 3–8 (1994). 7513193 10.1006/smim.1994.1002

[42] Liao X., Pirapakaran T., Luo X. M., Chemokines and chemokine receptors in the development of lupus nephritis. Mediators Inflamm. 2016, 6012715 (2016). 27403037 10.1155/2016/6012715 PMC4923605

[43] Crispin J. C., Kyttaris V. C., Terhorst C., Tsokos G. C., T cells as therapeutic targets in SLE. Nat. Rev. Rheumatol. 6, 317–325 (2010). 20458333 10.1038/nrrheum.2010.60 PMC2924434

[44] Bucy R. P., Xu X. Y., Li J., Huang G., Cyclosporin A-induced autoimmune disease in mice. J. Immunol. 151, 1039–1050 (1993). 8335890

[45] Prud’homme G. J., Parfrey N. A., Vanier L. E., Cyclosporine-induced autoimmunity and immune hyperreactivity. Autoimmunity 9, 345–356 (1991). 1954315 10.3109/08916939108997137

[46] Faulds D., Goa K. L., Benfield P., Cyclosporin. A review of its pharmacodynamic and pharmacokinetic properties, and therapeutic use in immunoregulatory disorders. Drugs 45, 953–1040 (1993). 7691501 10.2165/00003495-199345060-00007

[47] Venkatpurwar V. P., Rhodes S., Oienc K. A., Elliott M. A., Tekwe C. D., Jørgensen H. G., Kumar M. N. V. R., Drug- not carrier-dependent haematological and biochemical changes in a repeated dose study of cyclosporine encapsulated polyester nano- and micro-particles: Size does not matter. Toxicology 330, 9–18 (2015). 25637670 10.1016/j.tox.2015.01.017

