# Supplementary material for: A highly potent lymphatic system–targeting nanoparticle cyclosporine prevents glomerulonephritis in mouse model of lupus
Source: Sci Adv. 2020 Jun 12;6(24):eabb3900. doi: 10.1126/sciadv.abb3900 (PMC7292630; doi:10.1126/sciadv.abb3900)
Supplement: abb3900_SM.pdf [file abb3900_SM.pdf]

Science Advances
AAAS

advances.sciencemag.org/cgi/content/full/6/24/eabb3900/DC1

Supplementary Materials for

A highly potent lymphatic system–targeting nanoparticle cyclosporine prevents glomerulonephritis in mouse model of lupus

Raghu Ganugula, Meenakshi Arora, Dianxiong Zou, Sandeep K. Agarwal, Chandra Mohan, M. N. V. Ravi Kumar*

*Corresponding author. Email: mnvrkumar@tamu.edu

Published 12 June 2020, *Sci. Adv.* **6**, eabb3900 (2020)
DOI: 10.1126/sciadv.abb3900

The PDF file includes:

Figs. S1 to S6

Other Supplementary Material for this manuscript includes the following:

(available at advances.sciencemag.org/cgi/content/full/6/24/eabb3900/DC1)

Movies S1 to S5

Fig. S1. Highlights of major steps in polymer synthesis and nanoparticle formulation.

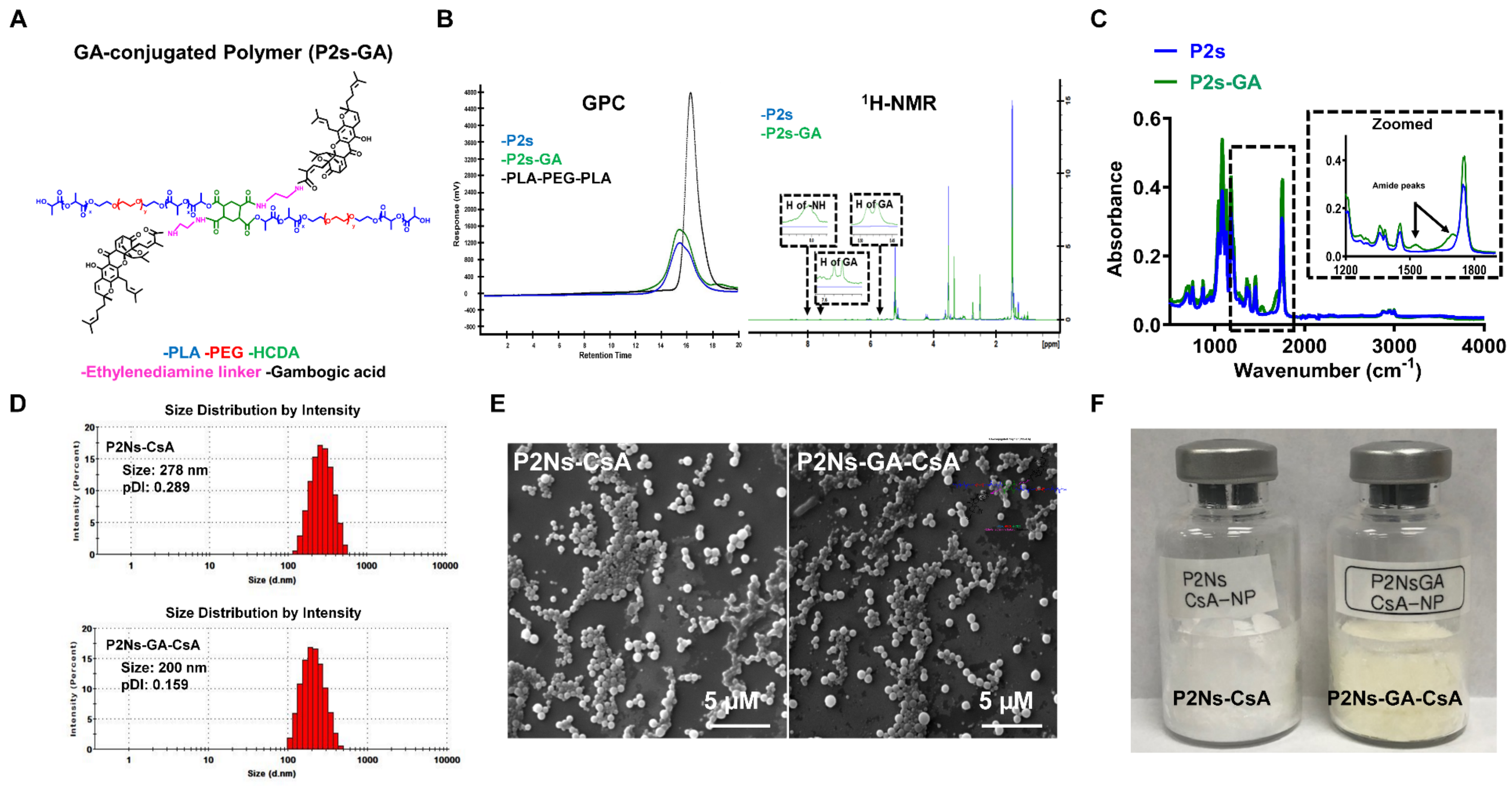

(A) Schematic illustration of the chemical structure of the P2s-GA polymer that assembles into P2Ns-GA; the constituents include: Polylactide (PLA, blue), polyethylene glycol (PEG, red), cyclohexanetetracarboxylic dianhydride (HCDA, green), ethylenediamine (pink), and gambogic acid (GA, black). **(B)** Polymers P2s (precursor of P2Ns) and P2s-GA (precursor of P2Ns-GA) were assayed by

gel permeation chromatography (GPC) to confirm size distribution, as well as by ^{1}H-NMR spectra of P2s and P2s-GA showing characteristic peaks attributing to the core polymer and to GA conjugation. **(C)** Fourier-transform infrared spectroscopy images of P2s and P2s-GA; note the additional amide peaks from ethylenediamine conjugation to the core polymer and GA. **(D)** Particle size distribution of P2Ns-CsA/P2Ns-GA-CsA were obtained by dynamic light scattering (DLS) as intensity (percent). Diameter and polydispersity index (pDI) of the nanoparticles were also shown. **(E)** Representative images from scanning electron microscopy of P2Ns-CsA/P2Ns-GA-CsA, (bar = 5 µm). **(F)** Representative photograph of P2Ns-CsA (left) and P2Ns-GA-CsA (right) freeze-dried formulations crimp-sealed in borosilicate vials. The particle sizes for fresh preparations of P2Ns-GA-CsA and P2Ns-CsA were between 160-197 nm, and the respective freeze-dried versions were 200-278 nm. While their zeta potentials values for fresh and freeze-dried versions were in the range of -38-41 and -27-37 mV respectively at pH 6-6.5 measured in deionized water. The slight variation between fresh versus freeze-dried particles could be due to aggregation coming from freeze drying process. All the *ex vivo/in vivo* studies were conducted with freeze-dried particles. (Photo Credit: Meenakshi Arora, Texas A&M University).

Fig. S2. Effect of CsA-void P2Ns-GA on SLE pathologies in MRL-*lpr* mice.

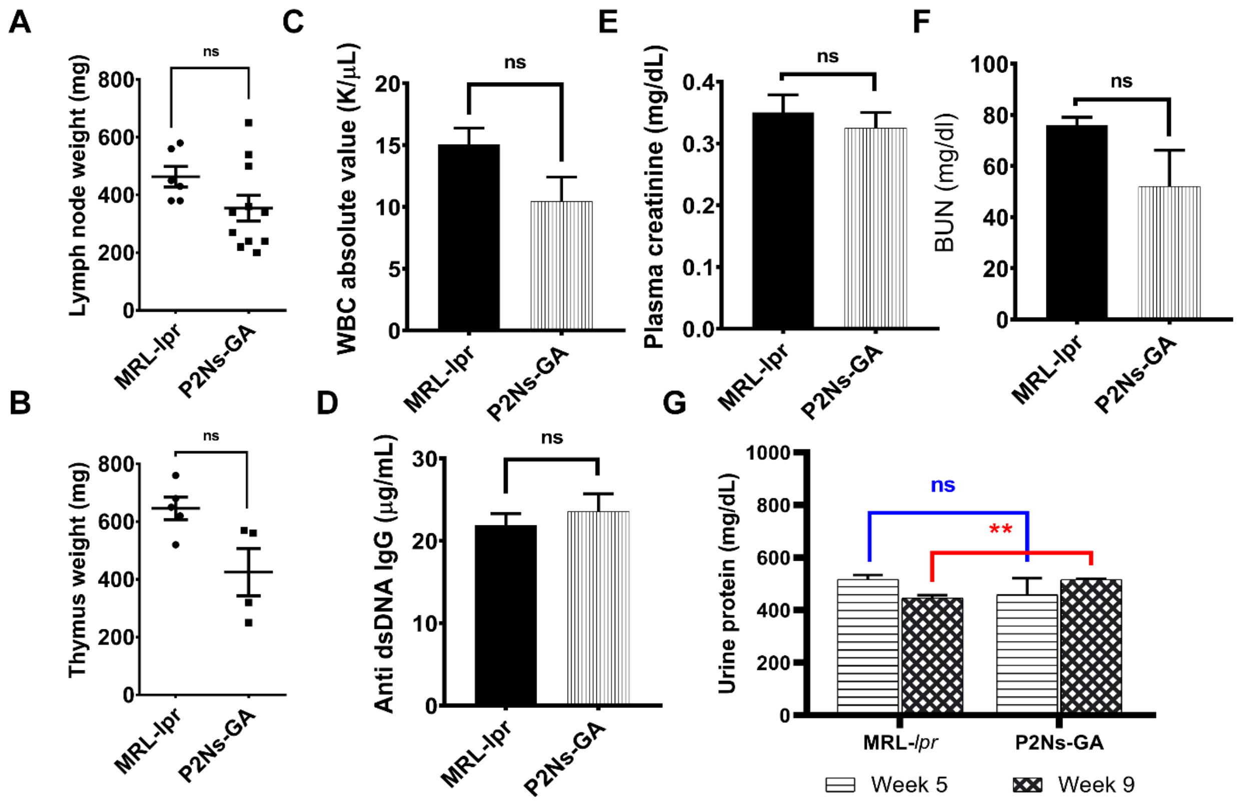

Female mice were treated with CsA-void, P2Ns-GA (equivalent to 5 mg/kg CsA particles) for 10 weeks, at three times weekly dosing. (**A)** Lymph node and (**B)** thymus weights compared to untreated MRL-*lpr* group (n=3-5 thymus; n=6-11 lymph nodes from 3-5 individuals). Blood panel of (**C)** WBC absolute count, (**D)** anti-dsDNA IgG, (**E)** plasma creatinine, (**F)** BUN, and of the P2Ns-GA group compared to MRL-*lpr* showing no measurable efficacy in any of the measured categories (n = 3 -5 individuals). (**G)** Urine protein were assayed from samples collected via hydrophobic sand at week 5 and 9 of treatment. Slight increases in urine protein was observed in the P2Ns-GA group between weeks 5 and 9 (n = 4 individuals). Data presented as mean ± SEM. $^{**}p < 0.001$. ns : not significant; statistics were conducted against the MRL-*lpr* group. Comparisons were made with one-way ANOVA followed by Tukey multiple comparison test.

Fig. S3. Analysis of lymphoid organ hypertrophy.

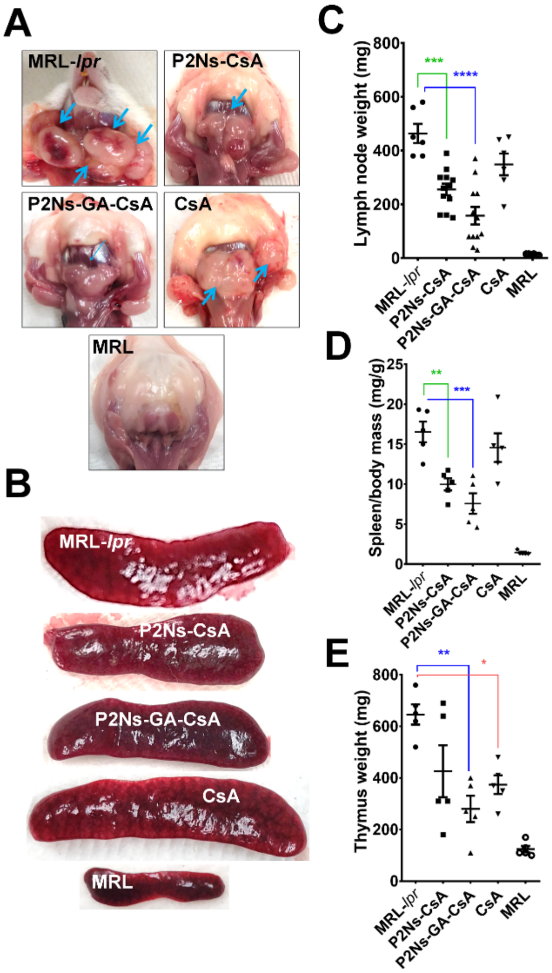

Lymphoid tissues from five groups of mice were analyzed post-euthanasia. (**A)** Significant lymphoadenopathy of cervical and axillary lymph nodes (blue arrows) and (**B)** splenomegaly were associated with untreated MRL-*lpr* mice. Graphical representations of (**C)** lymph node weight, (**D)** spleen/body mass, and (**E)** thymus weight measured from sacrificed mice (n = 5 individuals). *p < 0.05, **p < 0.01, ***p < 0.001, ****p < 0.0001. Statistics were conducted against MRL-*lpr* group. Comparisons were made with one-way ANOVA followed by Tukey multiple comparison test. (Photo Credit: Raghu Ganugula, Texas A&M University).

Fig. S4. Analysis of individual cytokine/chemokine change in SLE.

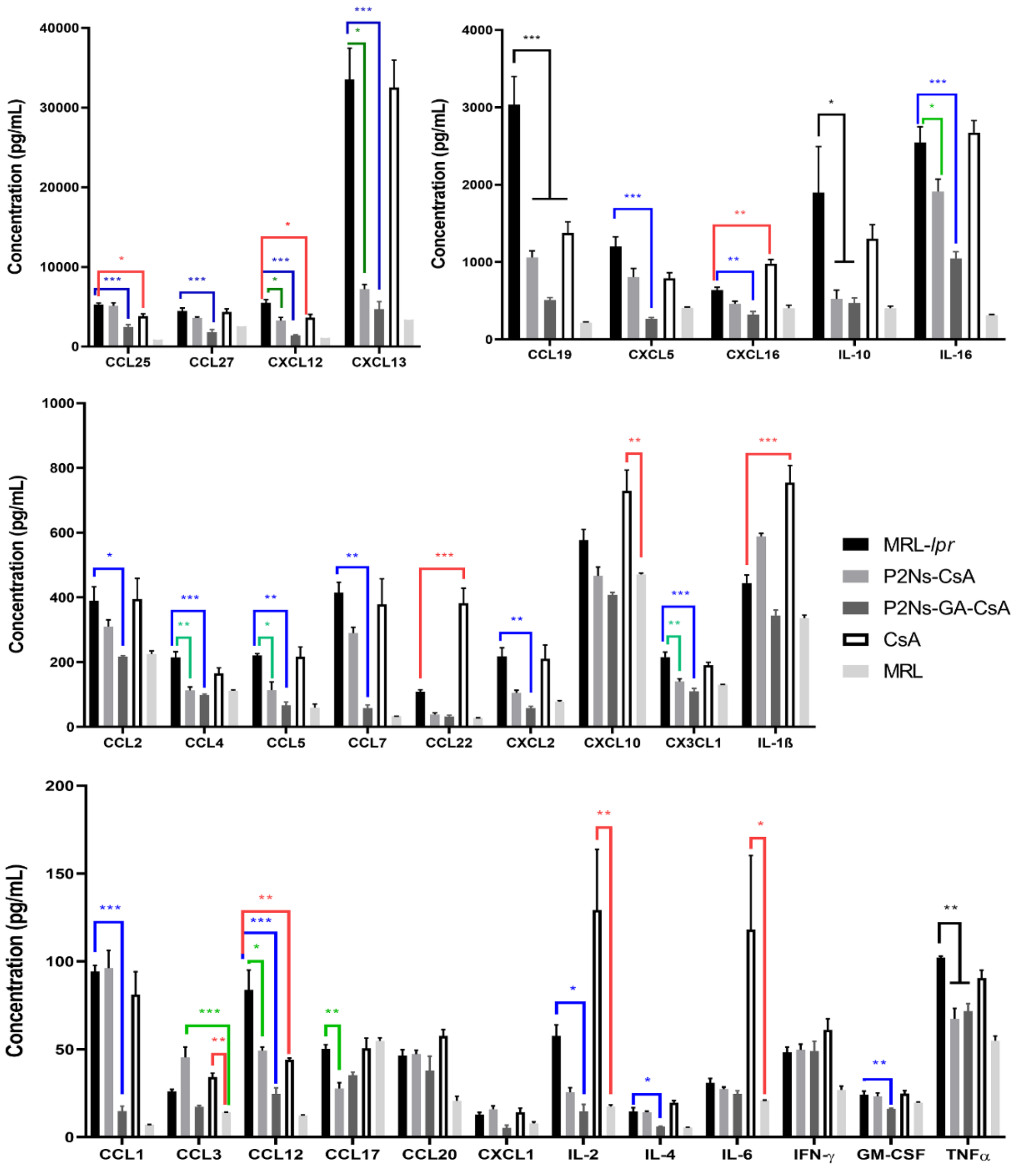

Graphical representations of cytokine/chemokine concentrations (pg/mL) detected in the plasma of each of the five experimental groups. Data for eotaxin, eotaxin-2, I-Tac, and TARC were not included because of high signal degradation and/or background noise from one of more samples

(n=3 individuals). *p < 0.05, **p < 0.01, *** p < 0.001. Data is presented as mean ±SEM. Comparisons were made with one-way ANOVA followed by Tukey multiple comparison test.

Fig. S5. Histological examination of lymphoid tissues.

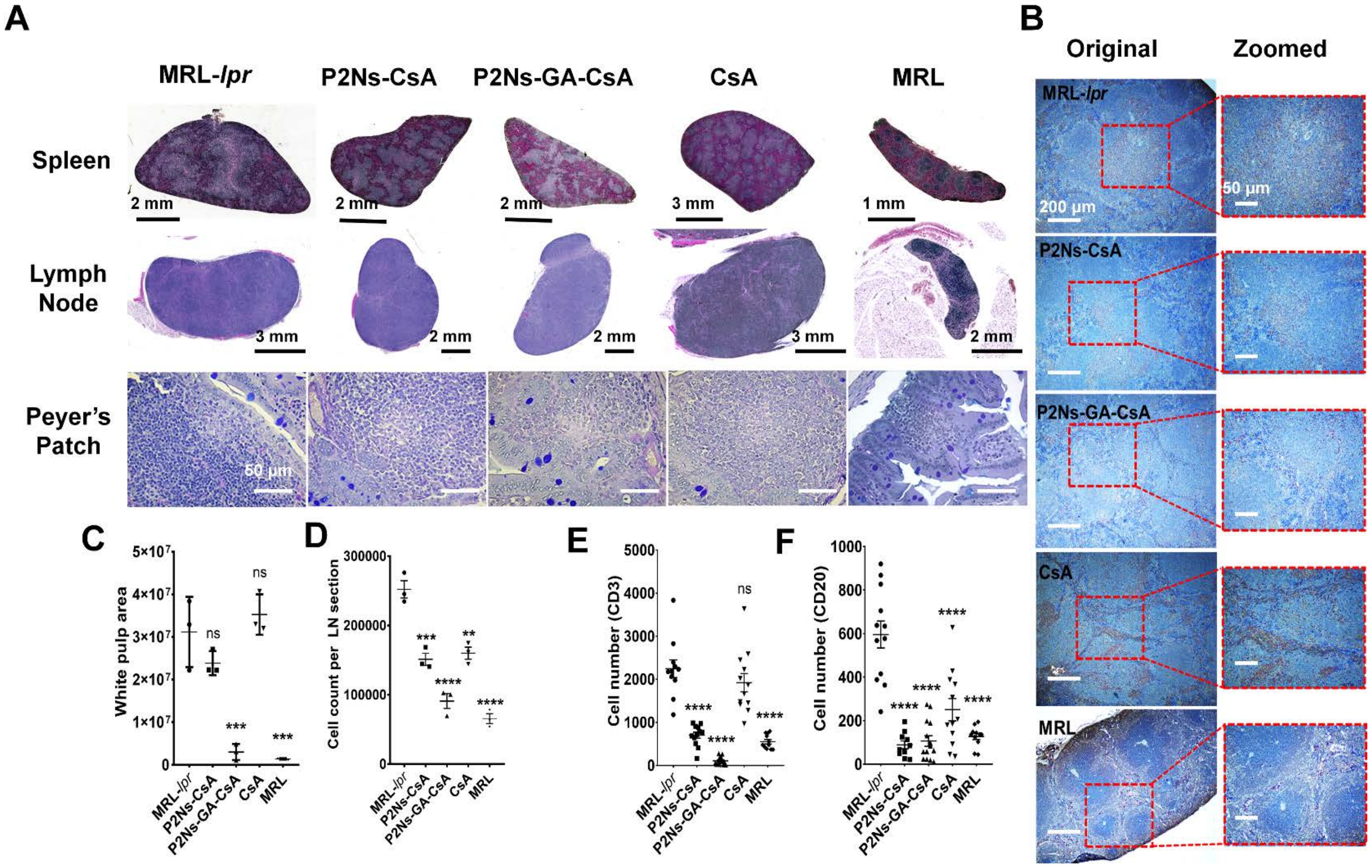

(A) Spleen, lymph node (LN), and Peyer's patch sections from five experimental groups. Spleen and LN analyzed with H&E stain; the final image was a h igh-resolution composite of 20-30 smaller micrographs. Peyer's patches were analyzed with periodic acid-

Schiff/alcian blue stain (Bar = 50 µm for all Peyer's patch micrographs). (**B)** Spleens were stained with anti-CD3 (brown) and anti-CD20 (red) antibodies and counterstained with hematoxylin to visualize lymphocytes. Large, punctate aggregates of $CD3^+$ and $CD20^+$ regions were observed in MRL-*lpr* and CsA groups, but the sizes of these regions were significantly reduced by P2Ns-GA-CsA (Bar = 200 µm for all original micrograph; bar = 50 µm for all zoomed micrographs). (**C)** Quantitation of white pulp (low H&E staining) area in the spleen. (**D)** Count of hematoxylin-staining regions in the LN section as a measurement of cell number. (**E)** Count of $CD3^+$ regions, and (**F)** count of $CD20^+$ regions in the spleen sections. Representative micrographs are shown; n = 15-30 images from at least 3 individuals. **p < 0.01, ***p < 0.001, ****p < 0.0001, ns: not significant. Statistics were conducted against the MRL-*lpr* group. Data is presented as mean ±SEM. Statistics were conducted against the MRL-*lpr* group. Comparisons were made with one-way ANOVA followed by Tukey multiple comparison test.

Fig. S6. Nephrin density evaluation in SLE glomeruli.

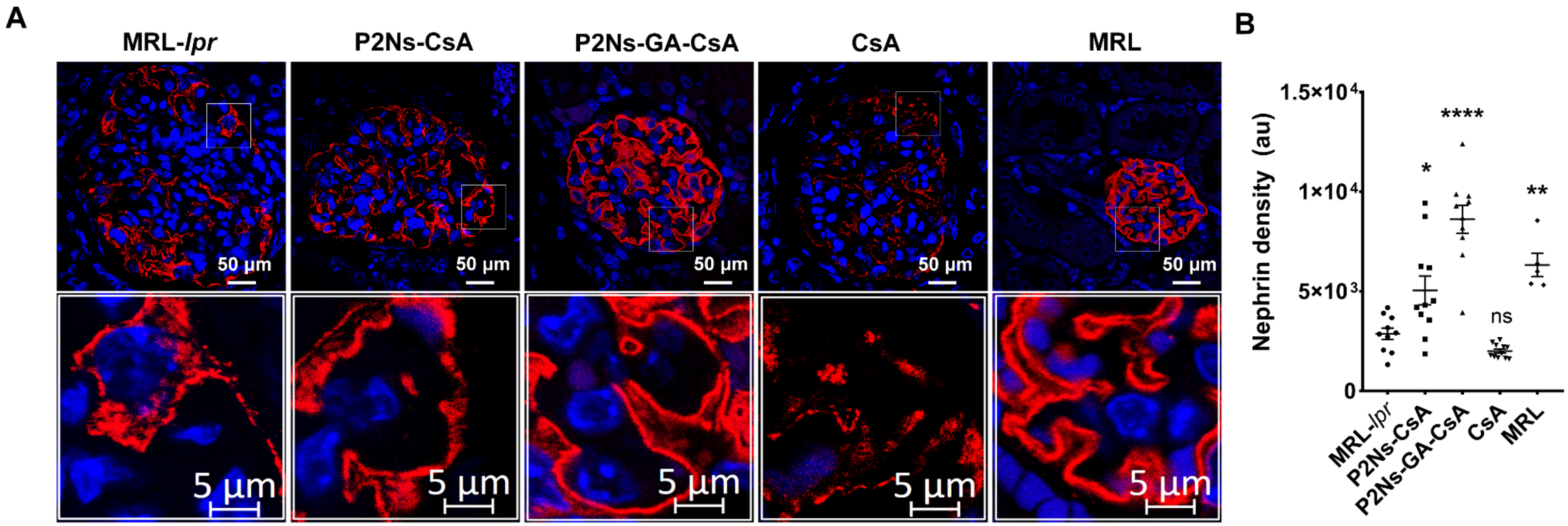

(A) Nephrin, the slit diaphragm component protein, was stained using anti-nephrin antibody (red) and counterstained using DAPI. The glomerular sections were imaged using confocal microscopy with 0.5 µm-thick z-slices for approximately 10 z-slices per section. (**B)** Nephrin density was calculated and graphed to show deterioration of slit diaphragm in MRL-*lpr* and CsA groups, coupled with treatment efficacy in P2Ns-GA-CsA. Representative micrographs are shown; n = 15-30 images from at least 3 individuals. Data presented as mean ± SEM. *p < 0.05, ****p < 0.0001. Statistics were conducted against the MRL-*lpr* group. Comparisons were made with one-way ANOVA followed by Tukey multiple comparison test.
